# Chemical synthesis of membrane proteins: a model study on the influenza virus B proton channel†
†Electronic supplementary information (ESI) available. See DOI: 10.1039/c8sc00004b


**DOI:** 10.1039/c8sc00004b

**Published:** 2018-01-22

**Authors:** A. C. Baumruck, D. Tietze, L. K. Steinacker, A. A. Tietze

**Affiliations:** a Darmstadt University of Technology , Clemens-Schöpf Institute of Organic Chemistry and Biochemistry , Alarich-Weiss Str. 4 , 64287 Darmstadt , Germany . Email: a.tietze@tietze-lab.de; b Darmstadt University of Technology , Eduard-Zintl-Institute of Inorganic and Physical Chemistry , Alarich-Weiss-Str. 4 , 64287 Darmstadt , Germany

## Abstract

In the present study we have developed and optimized a robust strategy for the synthesis of highly hydrophobic peptides, especially membrane proteins, exemplarily using the influenza B M2 proton channel (BM2(1–51)). This strategy is based on the native chemical ligation of two fragments, where the thioester fragment is formed from an oxo-ester peptide, which is synthesized using Fmoc-SPPS, and features an *in situ* cleavable solubilizing tag (ADO, ADO_2_ or ADO-Lys_5_). The nearly quantitative production of the ligation product was followed by an optimized work up protocol, resulting in almost quantitative desulfurization and Acm-group cleavage. Circular dichroism analysis in a POPC lipid membrane revealed that the synthetic BM2(1–51) construct adopts a helical structure similar to that of the previously characterized BM2(1–33).

## Abstract 2

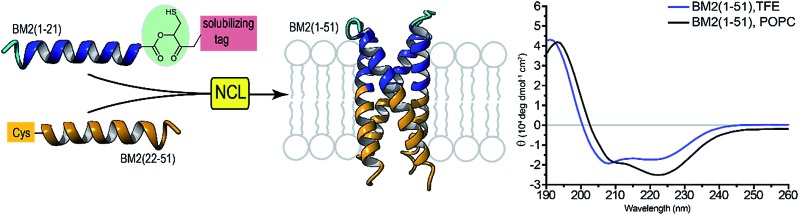
NCL results in the quantitative yield of a membrane protein, where a thioester peptide is formed from an oxo-ester with an *in situ* cleavable solubilizing tag.

## Introduction

Membrane proteins are key drug targets as they are involved in essential processes in the cell, including the control of information and material flow between cells and the mediation/propagation of nerve impulses.^1^–^4^ The study of membrane proteins has led to new and improved pharmaceutical treatments for a wide range of illnesses such as migraine, multiple sclerosis, and cancer, as well as muscle and immune system disorders. Ion channels, such as proton, sodium (Na_V_), potassium (K_V_) or calcium (Ca_V_) channels, are a type of membrane protein and are of immense importance in the human organism.^5^,^6^ The molecular characterization of membrane proteins is essential in order to understand their functional properties, and to elucidate the mode of action of potent drug leads. Despite intense efforts to gather structural information on some classes of membrane protein as well as their drug binding properties, providing enough material for such studies remains challenging.

The synthetic production of highly hydrophobic peptides, such as medium-sized fragments of membrane proteins representing functional parts, offers many advantages over other production strategies, such as protein expression, mainly because synthetic peptides can be customized and derivatized depending on the purpose of the research.^7^ Moreover, the production of multi-milligram amounts of membrane protein fragments by chemical synthesis might be even more important with respect to the development of bio-inspired materials.^8^

The syntheses of highly hydrophobic peptides are usually performed using specifically optimized synthetic strategies.^9^,^10^ Peptides with more than 50 amino acids in length are usually obtained by fusing two or several peptide fragments through native chemical ligation (NCL).^11^–^14^ The work up, including purification steps, for those peptides is usually challenging, since these peptides contain a high number of amino acids with hydrophobic side chains, which causes their aggregation in conventional solvents.^7^,^15^ The first attempt of the synthesis of influenza A virus M2 membrane protein was performed by Kochendoerfer *et al.* The authors used a Boc-based SPPS, and for the ligation step they used 6 M guanidinium chloride containing 20% TFE in order to solubilize the peptide, resulting in 65% product formation.^12^

Various methods have been established to overcome difficulties in the chemical synthesis of highly hydrophobic peptides, and they mainly focus on increasing the coupling efficiency^16^ by improving the protocols of Fmoc-based solid phase peptide synthesis (SPPS).^17^,^18^ The incorporation of pseudo-prolines was designed in order to reduce the aggregation during the chain prolongation by Fmoc-based SPPS, while at the same time being easily acid-cleavable.^19^,^20^ Alternatively, the incorporation of depsipeptide or *O*-acyl isopeptide units has been used to increase the solubility of hydrophobic peptides.^20^,^21^ However, even using these improved synthetic methods, purification remains extremely challenging. Aside from employing various mixtures of organic solvents^18^,^22^–^24^ and/or C4, C1 or phenyl columns^18^,^24^,^25^ for the HPLC purification of such peptides, the use of removable modifications,^26^–^32^ which can be incorporated to the peptide/protein’s C- or N-terminus or backbone, was demonstrated to partly overcome the problem of handling hydrophobic peptides during their production. Therefore, positively charged amino acids, such as polyarginines or polylysines, were employed in order to increase the solubility of hydrophobic peptides.^33^–^38^ However, these solubilizing tags are usually not removable and they alter the native sequence of the target peptide, which might impair peptide functionality. Consequently, removable solubilizing tags were developed which attach to the peptide *via* a cleavable linker (*e.g.* 4-hydroxymethyl benzoic acid (4-Hmb), 3,4-diaminobenzoic acid (Dbz) or 4-methoxy-5-nitrosalicylaldehyde),^30^,^39^ an amino acid side chain/backbone,^28^,^29^,^40^ or a short peptidase recognition site (which can be cleaved after the acidic residues using carboxypeptidase B).^36^,^39^,^41^,^42^ Nevertheless, only a few examples of cleavable solubilizing tags for chemical synthesis are described to date (Table S1†).

Considering the NCL mechanism, the addition of solubilizing tags on the thioester-leaving group might be another promising strategy to increase the solubility of hydrophobic peptides, since the solubilizing tag is removed during the process of ligation, retaining the native peptide sequence. In 2007, Johnson *et al.* incorporated a polyarginine (Arg6) solubilizing tag attached to a thioester-leaving group using a Boc-based peptide synthesis protocol (Table S1†).^33^ Most of the examples described in the literature for the synthesis of membrane proteins and their parts were by Boc-SPPS (Table S1†), which usually requires special equipment, due to the use of HF in the final global deprotection and cleavage from the resin used in SPPS.^43^,^44^ Although, some Fmoc-based methods using peptide hydrazides were applied for membrane protein synthesis.^13^,^45^ However, incorporation of a C-terminal solubilizing tag into peptide hydrazides is problematic. Therefore, a solution for this problem had to be found.

About a decade ago, an interesting Fmoc-SPPS compatible oxoester-based NCL strategy was described, which relied on the *in situ* generation of the thioester through an *O*-to-*S* acyl shift of the 2-hydroxy-3-mercaptopropionic acid (Hmp) moiety, which then reacts with the Cys-fragment to generate the final ligation product.^46^–^48^ Using this method, small glycopeptides,^47^ the NNY-Rantes polypeptide chain (comprising residues 1–68),^49^ and cyclotides^50^ have been synthesized. However, under standard NCL conditions, high amounts (20–25%) of the hydrolyzed Cα-carboxyester by-product were reported.^49^,^51^ Moreover, the Hmp unit was found to be labile during standard Fmoc-SPPS, which might explain why this elegant strategy has not found a wide use. In 2013, Liu *et al.* observed that the use of 2-methylpiperidine instead of piperidine for the Fmoc-deprotection step nicely solved the instability problem during the Fmoc-SPPS of peptide-oxoesters.^51^

At this point, we realized that this strategy can be employed to introduce solubilizing tags during Fmoc-SPPS that can be easily cleaved in a one-step reaction during the NCL procedure, resulting in a significant improvement of the NCL of highly hydrophobic protein fragments. Consequently, the thioester-forming Hmp unit would serve as a cleavable linker between the solubilizing tag and the peptide chain.

Thus, our strategy combines the ligation of a conventional Cys-peptide fragment to an N-terminal fragment, which carries a removable, thioester-forming Hmp unit followed by a short PEG linker and a solubilizing tag. To demonstrate the feasibility of our anticipated strategy, we sought to synthesize small model peptides and an extended part of the membrane region of the influenza B proton channel (BM2), which is a highly hydrophobic peptide and is prototypical for the class of small membrane-spanning ion channels. Moreover, BM2 represents an important drug target for the treatment of seasonal flu, and its molecular structure has not been fully solved yet.^52^–^55^ An efficient synthesis of such ion channel-forming peptides is of high importance, especially to the NMR community and their need for isotopically labelled samples.

## Results and discussion

### Development and optimization of the synthesis and NCL protocol for the Hmp unit using model peptides

**Fig. 1:**
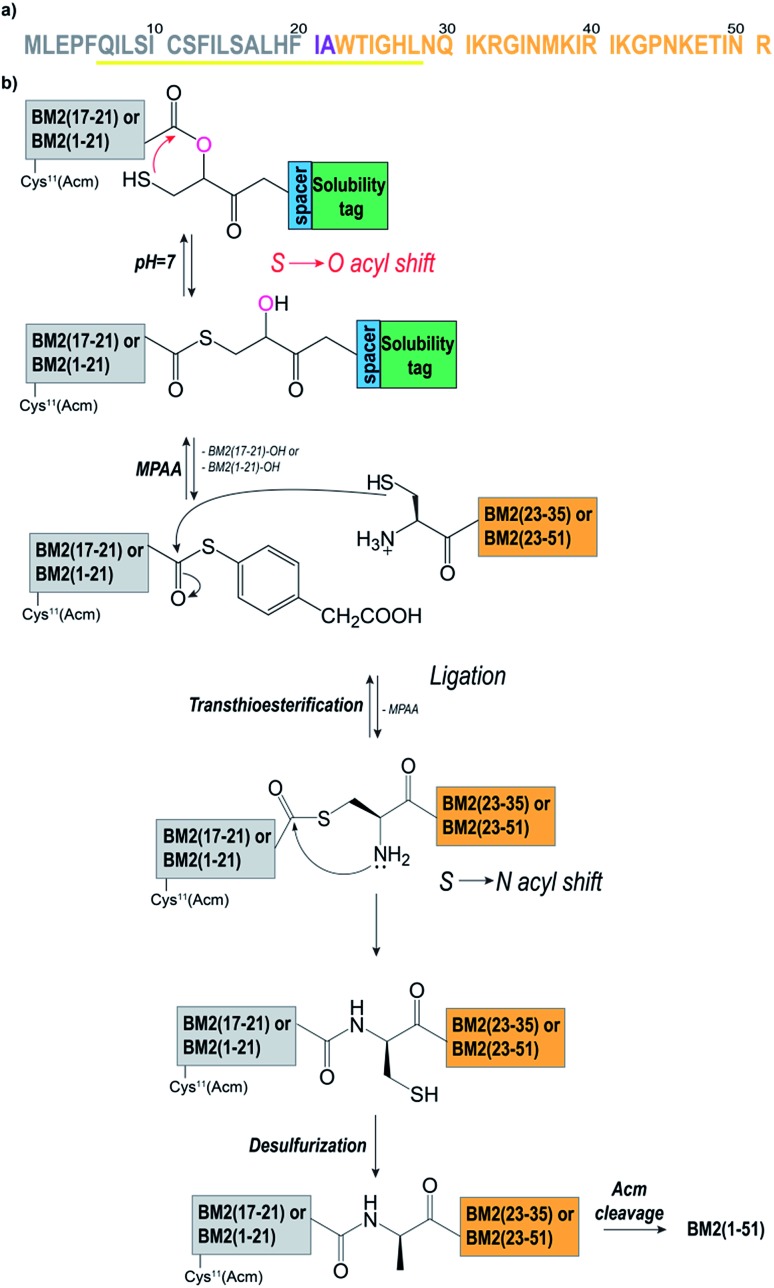
(a) The amino acid sequence of BM2(1–51). The membrane spanning helical region is highlighted in yellow. Residues Ile21 and Ala22 were mutated to Leu and Cys respectively. The Hmp-fragments [Cys^11^(Acm)]BM2(1–21)/BM2(17–21) are coloured in grey. The Cys-fragments [Cys^22^]BM2(22–51)/[Cys^22^]BM2(22–35) are coloured in orange. (b) The NCL protocol as applied for the synthesis of BM2(1–51). The Hmp-fragment BM2(17–21) or [Cys^11^(Acm)]BM2(1–21) formed the thioester at pH 7 *via* an *S* to *O*-acyl shift followed by attachment of the Cys-fragment [Cys^22^]BM2(22–35) or [Cys^22^]BM2(22–51). Simultaneously, the cleavage of the solubilizing tag occurred. Then, the MPAA-catalysed transesterification was followed by an irreversible *S* to *N*-acyl shift. Finally, Cys22 was converted to Ala22 through desulfurization, and the Acm protecting group was cleaved from Cys11.

In order to optimize the synthetic strategy for the Hmp-peptide fragments (the thioester-forming peptide), a set of four different model peptides (**1–4**) was employed and ligated to a small Cys-peptide fragment (**5**) (Table S2 and S3,† and Fig. 1). The sequences of these peptides were derived from the influenza virus BM2 protein sequence (Fig. 1a, Table S2†), comprising residues 17 to 21 for the Hmp-peptide and residues 22 to 35 for the Cys-peptide fragment.

As described above, the Hmp unit was attached to a polylysine and/or mini-PEG solubilizing tag and incorporated into the model peptide fragments **1–4**. More precisely, the thioester-forming peptides **2–4** were composed of four consecutive units: the peptide sequence, the Hmp unit,^49^,^51^ a spacer (if necessary), and the solubilizing tag (Fig. 1b). Peptide **1** did not contain any solubilizing tag and/or spacer, and was used as a reference. During the NCL reaction, the Hmp unit forms the required intermediate thioester through an intermolecular *S* to *O*-acyl transfer, which is then followed by cleavage of the solubilizing tag upon formation of the MPAA thioester (Fig. 1b). Prior to the synthesis of the model peptides **1–4**, the Hmp unit was synthesized as a racemic mixture according to the procedure described by K. Wisniewski.^56^ The Hmp racemic mixture was directly used for Fmoc-SPPS, leading to a peptide mixture containing diastereomer A and diastereomer B which eluted in HPLC chromatograms at different retention times.

In the first attempt, the native BM2 sequence comprising residues 17 to 21 (ALHFI, Fig. S3a, b†) was coupled to the Hmp unit, resulting in a very low ligation yield and a high degree of carboxyester hydrolysis (90%) (Fig. S1c†). This result was caused by the poor *S* to *O*-acyl shift yields when isoleucine (Ile21) is the C-terminal amino acid, as reported by Liu *et al.*^51^ Therefore, Ile21 was mutated to Leu21, which did not show such a high degree of carboxyester hydrolysis.^51^ Moreover, Ala22 was chosen as the respective ligation point, and was thus changed to Cys22 (Fig. 1a).

Polylysine (Lys_5_)^57^,^58^ or one or two 8-amino-(ADO) units were tested as solubilizing tags.^59^ Additionally, ADO was used as a spacer in combination with the Lys_5_ tag, in order to reduce any steric hindrance arising from the lysine Boc-protecting groups during the Hmp coupling (Table S2†).

The thioester-forming peptides ALHFL-Hmp (**1**), ALHFL-Hmp-ADO (**2**), ALHFL-Hmp-ADO_2_ (**3**), and ALHFL-Hmp-ADO-Lys_5_ (**4**) were obtained as Hmp-diastereomers in yields between 20–60% (Table S2, Fig. S4, S5†). The crude peptides **1–3** were directly subjected to the ligation experiments (Fig. S4a–c†), whereas peptides **4–5** were first purified by RP-HPLC (Fig. S4d, S5†). Two dipeptides were synthesized in order to analyse whether the first amino acid (l-Leu), which is coupled to Hmp *via* the Mitsunobu reaction,^60^ is prone to racemization: l-Phe-l-Leu-Hmp and l-Phe-d-Leu-Hmp. HPLC analysis revealed different retention times for both dipeptides upon coelution, indicating that l-Leu does not racemize upon coupling to the racemic Hmp (Fig. S4e, f†). The desired peptides were stored at –20 °C, and neither degradation nor hydrolysis was observed. This is a significant advantage of our HMP-thioester strategy in comparison to methods where thioester-peptides, which are unstable at higher pH, are synthesized in a multistep reaction (*i.e.* on 4-sulfamylbutyryl resin).^61^

**Fig. 2:**
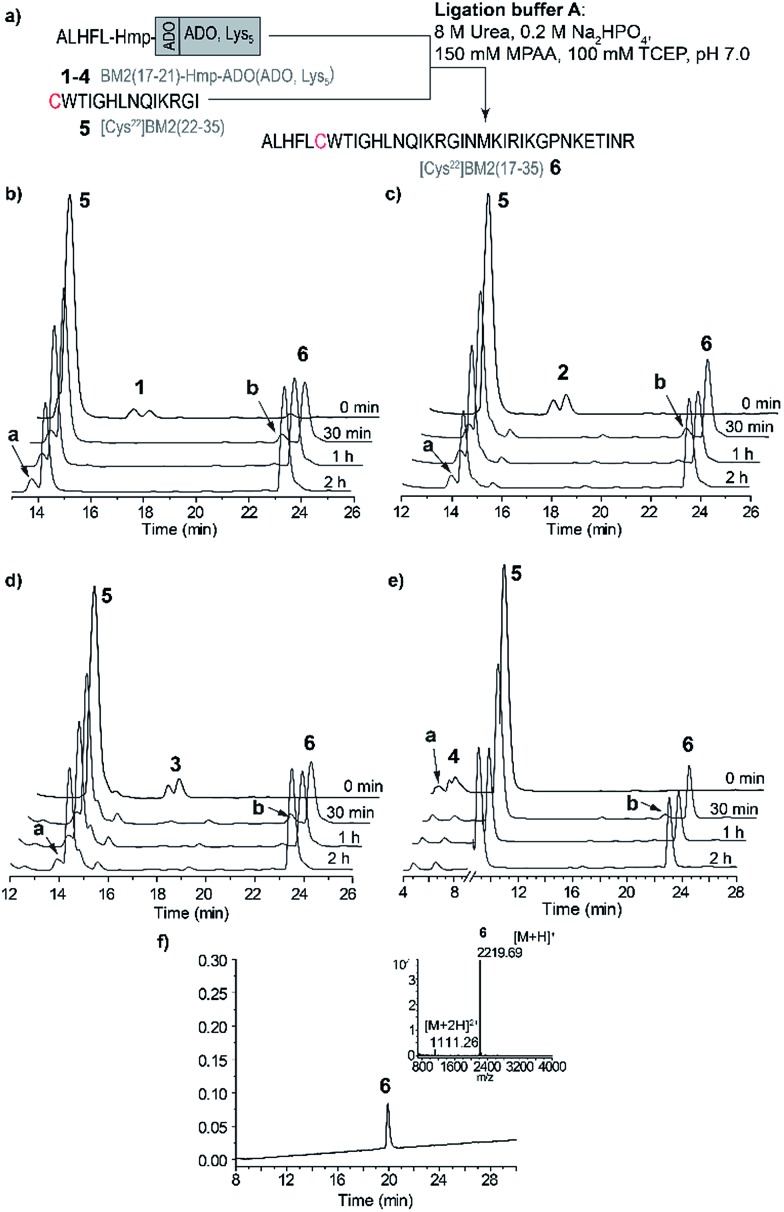
(a) A schematic representation of the NCL strategy in ligation buffer A for the model peptides. (b–e) RP-HPLC elution profiles of the model peptide ligations: (b) ALHFL-Hmp (**1**), (c) ALHFL-Hmp-ADO (**2**), (d) ALHFL-Hmp-ADO_2_ (**3**) and (e) ALHFL-Hmp-ADO-Lys_5_ (**4**) with the Cys-fragment BM2(22–35) (**5**). The MPAA peak was removed from the chromatogram for better visibility. (f) RP-HPLC of the purified ligation product BM2(17–35) (**6**) and ESI-MS spectrum (inset). HPLC conditions: (b–d) 15–45% eluent B over 30 min, (e) 20–40% eluent B over 30 min and (f) 15–45% eluent B over 30 min. The hydrolyzed ester BM2(17–21)-CαOOH is indicated by **a** in the elution profiles, and BM2(17–21)-MPAA thioester is indicated by **b**.

The model peptides **1–4** were ligated to the Cys-fragment BM2(22–35) (**5**) at a molar ratio of 1 : 2 in ligation buffer A, and were very soluble under these conditions (Table S6†). We noticed already at this step that peptide **4**, possessing the Lys_5_ solubilizing tag attached to the Hmp unit, is the least hydrophobic, eluting at ∼22% of eluent B (acetonitrile containing 0.1% TFA). This is in comparison to peptides **1–3**, which all elute at ∼32% of eluent B, and possess ADO, ADO_2_ or no solubilizing tag (Fig. 2a–d). Consequently, ADO and ADO_2_ did not influence the solubility of the respective model peptide in comparison to the reference peptide **1**.

The ligations for the model peptides **1–4** were completed within 2 hours (Fig. 2), and no further changes were observed at longer reaction times (>2 h), according to the RP-HPLC analysis. For peptide **1**, the BM2(17–21)-MPAA thioester, which is generated through the *in situ* rearrangement of Hmp and reaction with MPAA, was already present directly after starting the ligation. However, for peptides **2–4** the maximum amount of the thioester was formed at 30 min (Fig. 2b). The newly formed thioester-intermediate was subsequently converted into the MPAA-thioester, which was then ligated through transthioesterification with the C-terminal Cys of the BM2(22–35) (**5**) fragment. After the *S* to *N*-acyl shift, the final peptide ALHFLCWTIGHLNQIKRGI (BM2(17–35) (**6**)) (Fig. 1b and 2d) was obtained in 80–90% yield, according to the HPLC data (Table S3†).

As already mentioned above and in accordance with the literature, the only byproduct formed during the ligation process was from hydrolysis of the carboxyester.^49^,^51^ According to the RP-HPLC analysis, 13–16% of the side product ALHFL-CαOOH was formed during the ligation reactions of **1–4** with **5** (Table S3,† Fig. 2), which is comparable to the literature data.^49^,^51^

However, the final yields of the product **6** during the ligation reaction of the peptides **1–4** with **5** were similar, ranging from 80–90%. Hence, the introduction of a solubilizing tag into the model peptide **1**, resulting in the peptides **2** (ADO), **3** (ADO_2_) and **4** (ADO-Lys_5_), did not impair the efficiency of the ligation reaction. Furthermore, our results indicate reaction times of less than 2 h for the NCL of **1–4** with **5**.

### Application of the NCL strategy for the synthesis of BM2(1–51)

Encouraged by the results for the NCL of the model peptides **1–4** with **5**, we decided to apply this strategy to longer and more hydrophobic peptides. Hence, we intended to synthesize a BM2 fragment which comprises residues 1–51, and now includes the full transmembrane domain and parts of the cytosolic part of the M2 protein. As for the model peptides **1–4**, Ala22 was chosen as the respective ligation point, and thus mutated to Cys22 (Fig. 1). Moreover, the thiol of Cys11 was protected with Acm in order to prevent a possible cyclization reaction (*viz.* reaction of the thiol group with the thioester) during the NCL. In analogy to the NCL strategy for the model peptides, ADO_2_ and Lys_5_ solubilizing tags were employed in combination with the Hmp unit (**9–10**, Table S2, Fig. S6†) for the thioester-forming peptide [Cys^11^(Acm)]BM2(1–21). Additionally, we synthesized BM2(1–21) (**7**) and [Cys^11^(Acm)]BM2(1–21)-Hmp (**8**) as references.

Peptides **7–11** were synthesized as described earlier for the model peptides. For peptides **8–10**, 2-methylpiperidine (2-MP) was used for Fmoc-deprotection to avoid premature peptide cleavage from the resin.^51^

In the following sections, the NCL of the peptides **8–10** with the peptide fragment **11** was performed in three different ligation buffers, namely phosphate buffer (named buffer from hereon), buffer/trifluoroethanol (2 : 1, v/v) and buffer/hexafluoro-2-propanol (2 : 1, v/v).

### Ligation reaction in phosphate buffer (buffer A)

**Fig. 3:**
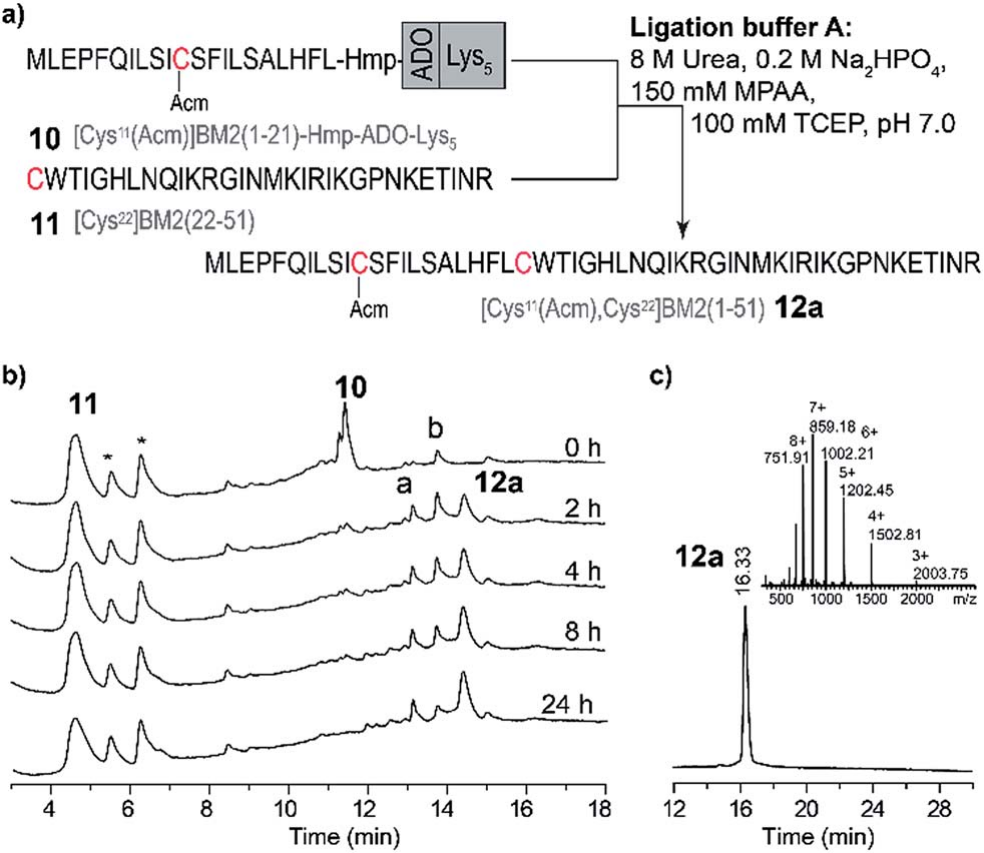
Ligation of [Cys^11^(Acm)]BM2(1–21)-Hmp-ADO-Lys_5_ with [Cys^22^]BM2(22–51) in the ligation buffer A. (a) Schematic representation of the NCL strategy in the ligation buffer A. (b) RP-HPLC chromatograms of the reaction mixture at different times. RP-HPLC conditions: 10–20% eluent B over 5 min followed by 20–70% eluent B over 20 min. (c) RP-HPLC of the purified ligation product [Cys^11^(Acm), ^22^Cys]BM2(1–51) (**12a**) and the ESI-MS spectrum (inset). The hydrolyzed ester [Cys^11^(Acm)]BM2(1–21)-C^α^OOH is labelled with **a**. [Cys^11^(Acm)]-BM2(1–21)-MPAA thioester is labelled with **b**.* – peaks from the ligation solution.

Due to the high hydrophobicity of the thioester forming [Cys^11^(Acm)]BM2(1–21) peptide fragment, peptides **8** and **9** were not soluble in the standard ligation buffer, disallowing the NCL of these peptides. In contrast, a significant improvement of the solubility was achieved for the peptide [Cys^11^(Acm)]BM2(1–21)-Hmp-ADO-Lys_5_ (**10**), allowing for an efficient ligation with the Cys-fragment [Cys^22^]BM2(22–51) (**11**) (Fig. 3) under standard ligation conditions in phosphate buffer (ligation buffer A).

In analogy to the model peptides, the [Cys^11^(Acm)]BM2(1–21)-MPAA thioester was formed directly after the reaction was started (Fig. 3), indicating that the MPAA-thioester formation does not depend on the peptide length. Interestingly, the ligation reaction took much longer to complete compared to the model peptides, almost fully converting the [Cys^11^(Acm)]BM2(1–21)-MPAA thioester into the ligation product [Cys^11^(Acm), Cys^22^]BM2(1–51) (**12a**) after 24 h (Fig. 3). In comparison to the ligation of the model peptide ALHFL-Hmp-ADO-Lys_5_**4**, the amount of hydrolyzed carboxyester [Cys^11^(Acm)]BM2(1–21)-C^α^OOH was nearly the same (20%), resulting in an overall 80% yield of the desired product [Cys^11^(Acm), Cys^22^]BM2(1–51) (**12a**) (Table S3 and S4†). In summary, our ligation conditions and the proposed synthesis of the thioester-forming fragment, possessing a combination of solubilizing tags (Lys_5_) and the thioester-forming Hmp unit, result in high yields (80%) of the desired product, and can be used generally for the NCL of highly hydrophobic peptide thioester-fragments. Even though our data already indicated a significant improvement for the NCL of hydrophobic peptides, we sought to further improve the anticipated strategy by suppressing the carboxyester formation. Therefore, we used 2,2,2-trifluoroethanol (TFE) or hexafluoro-2-propanol (HFIP), which were added to the ligation buffer A in a 1 : 2 (v/v) ratio, resulting in the ligation buffer B or C (Table S6†), respectively. TFE was previously described as an efficient additive of the ligation reaction for the synthesis of the M2 protein from influenza A virus.^12^,^62^

### Ligation reaction in trifluoroethanol/phosphate buffer (buffer B)

**Fig. 4:**
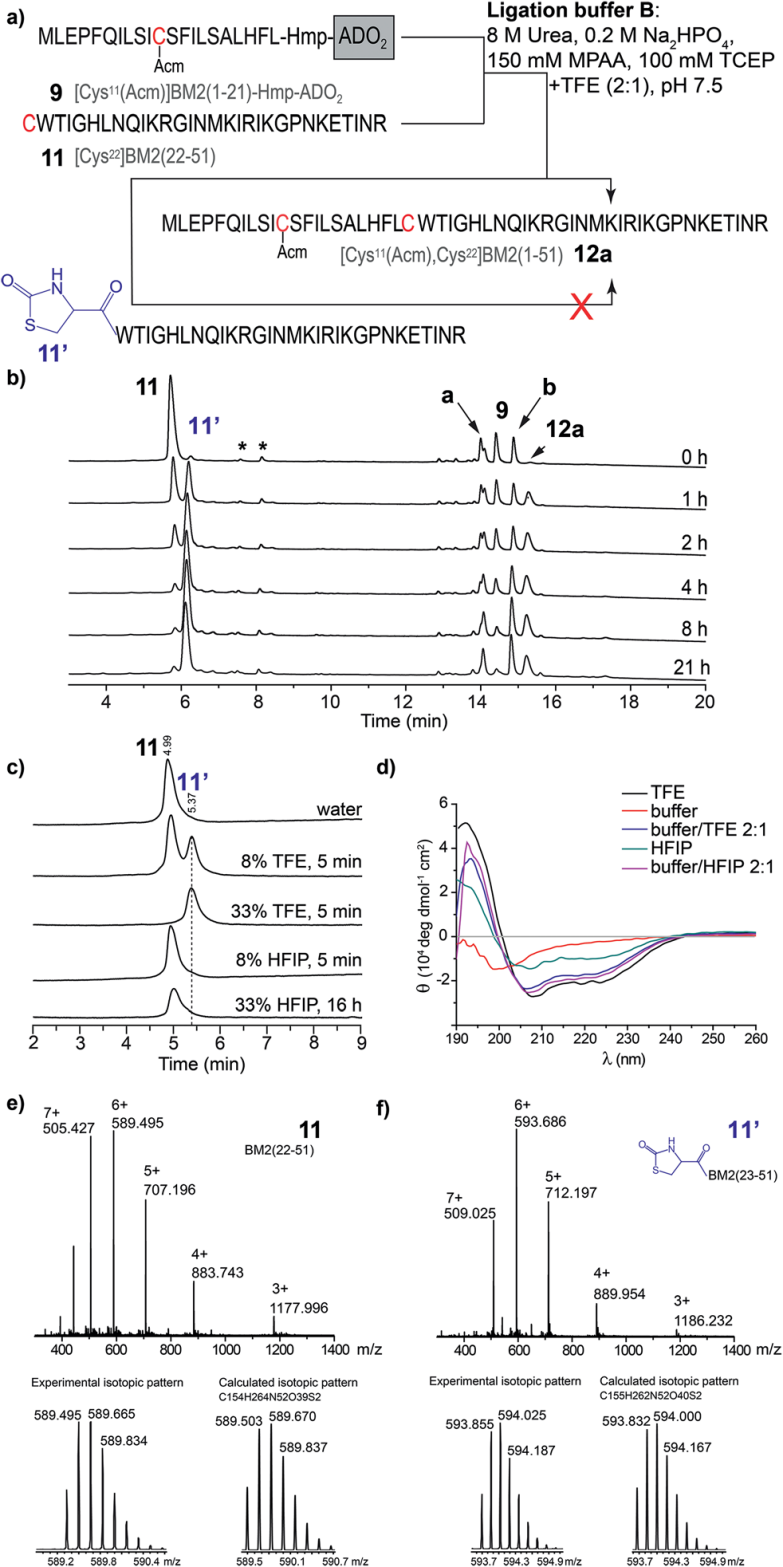
(a) Schematic representation of the NCL in the ligation buffer B. (b) RP-HPLC chromatograms of the ligation reaction mixture of [Cys^22^]BM2(22–51) with [Cys^11^(Acm)]BM2(1–21)-Hmp-ADO_2_ (**10**). The hydrolyzed ester [Cys^11^(Acm)]BM2(1–21)-C^α^OOH is indicated as **a**. The [Cys^11^(Acm)]BM2(1–21)-MPAA thioester is indicated as **b**. Peaks which result from the ligation buffer are indicated with *. HPLC conditions: 10–20% eluent B over 5 min followed by 20–70% eluent B over 15 min at a flow rate of 2 mL min^–1^. (c) HPLC analysis of [Cys^22^]BM2(22–51), which was incubated in different solutions. HPLC conditions: 10–40% eluent B over 15 min at a flow of 2 mL min^–1^. (d) CD spectra of [Cys^22^]BM2(22–51) recorded in different solutions. (e) MS spectrum of [Cys^22^]BM2(22–51) **11** (*t*_R_ 5.71 min), and experimental *vs.* calculated isotopic pattern. (f) MS spectrum of [Cys^22^]BM2(22–51) **11′** (*t*_R_ 5.78 min), and experimental *vs.* calculated isotopic pattern.

In contrast to the NCL under standard conditions using the phosphate buffer (buffer A), the thioester-forming fragments **8–10** and Cys-fragment **11** were highly soluble in the TFE-containing ligation buffer B. However, during the NCL of **9** and **11** (Fig. 4a), the product formation of [Cys^11^(Acm), Cys^22^]BM2(1–51) (**12**) seemed to be completed after 2 h, while the amount of the hydrolyzed carboxyester ([Cys^11^(Acm)]BM2(1–21)-C^α^OOH) continuously increased throughout the entire reaction process. Interestingly, the amount of the Cys-fragment [Cys^22^]BM2(22–51) (**11**, *t*_R_ 5.71 min) continuously decreased, with the continuous formation of an unknown side product (**11′**) observed at *t*_R_ 5.78 min (Fig. 4a). As concluded from the HPLC chromatograms, this side product appears to be unreactive towards the formation of the ligation product **12**. A detailed analysis of this newly formed side product, using HPLC, ESI-MS and CD spectroscopy, revealed that **11′** directly resulted from **11**, presumably induced by TFE (Fig. 4).

This interpretation is further supported by the fact that **11′** was directly formed in an aqueous TFE solution. In contrast, peptide **11** was totally stable in an aqueous HFIP solution, concluding that the strong helix-inducing effect of TFE and HFIP is not responsible for the formation of **11′**, as further indicated by the CD spectra shown in Fig. 4d. Finally, MS spectrometry uncovered a difference of *m*/*z* = 26 when comparing **11′** to **11**, indicating a TFE-induced chemical modification (Fig. 4e and f), presumably with formation of a 2-oxo-4-thiazolidine with the N-terminal Cys.^63^,^64^ In 1987, D’Ischia *et al.* described the reaction of aminothiols with 1,1′-carbonyldiimidazole, resulting in the formation of 2-oxo-4-thiazolidines.^63^ The TFE-induced formation of a 2-oxo-4-thiazolidine nicely explains the inactivation of **11** under the NCL conditions and the difference in *M*_w_ of *m*/*z* = 26. We suggest that urea would act in the same way as 1,1′-carbonyldiimidazole in this reaction, providing the carbonyl group at the 2 position of the thiazolidine ring. The calculated isotopic pattern for this suggested 2-oxo-4-thiazolidine modified [Cys^22^]BM2(22–51) is in good agreement with the experimental isotopic pattern (Fig. 4f). Moreover, the formation of such a non-reactive side product nicely explained the rather high amount of the hydrolyzed carboxyester (∼45%) under these conditions, since the Cys-fragment is not available for the ligation reaction, and the thioester fragment hydrolyses to a great extent. In particular, the reaction mechanism was not analyzed further, since we found that HFIP is a good alternative to TFE during the NCL, without forming any unreactive product with the peptide.

### Ligation reaction in hexafluoro-2-propanol/phosphate buffer (buffer C)

**Fig. 5:**
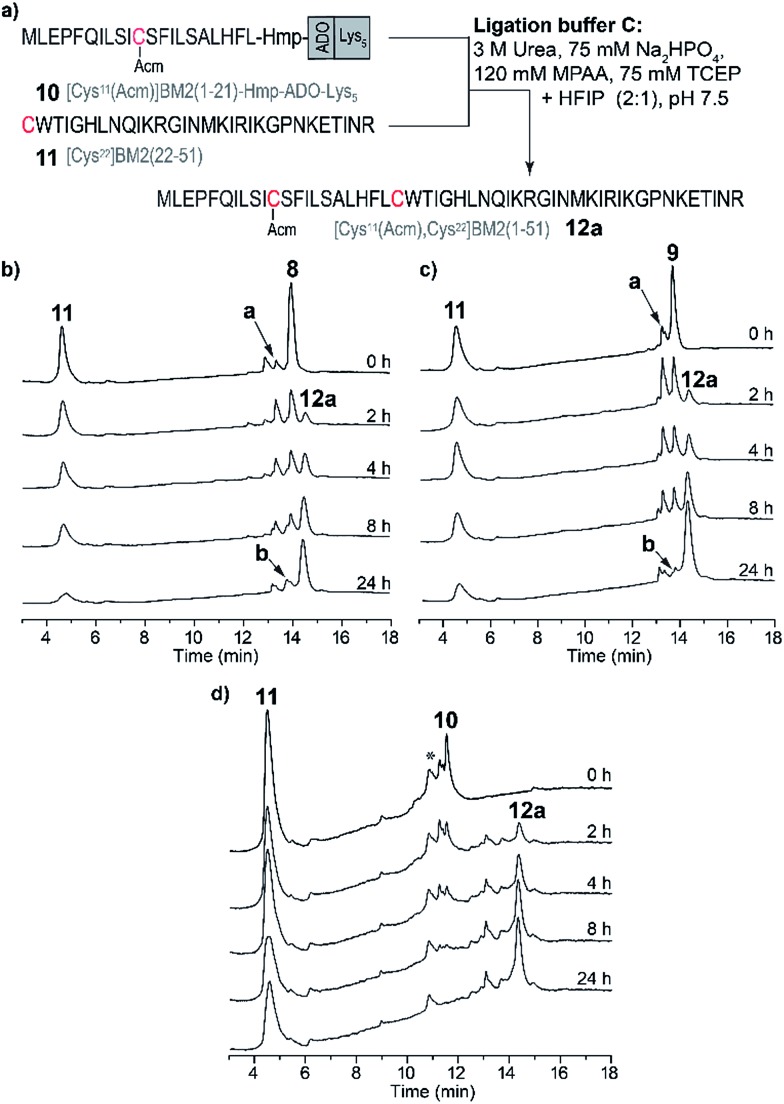
(a) Schematic representation of the NCL in the ligation buffer C. RP-HPLC chromatograms of the ligation reaction mixtures of the Cys-fragment [Cys^22^]BM2(22–51) (**11**) with (b) [Cys^11^(Acm)]BM2(1–21)-Hmp (**8**), (c) [Cys^11^(Acm)]BM2(1–21)-Hmp-ADO_2_ (**9**) and (d) [Cys^11^(Acm)]BM2(1–21)-Hmp-ADO-Lys_5_ (**10**). Hydrolyzed carboxyester [Cys^11^(Acm)]BM2(1–21)-C^α^OOH is highlighted as **a**. [Cys^11^(Acm)]BM2(1–21)-MPAA thioester is highlighted as **b**. Some impurities on the HPLC chromatogram (d) are highlighted with *.

Because of the stability of **11** in HFIP (Fig. 4c), a third ligation buffer system (Table S6†) was tested. Due to the reduced solubility in HFIP of the ligation additives TCEP, MPAA and urea, they were used in slightly lower concentrations (Fig. 4 and 5). As outlined in Fig. 5, the NCL of the peptide fragments **8–10** with **11** was completed within 24 h, almost quantitatively forming the product **12a**. For [Cys^11^(Acm)]BM2(1–21)-Hmp (**8**) and [Cys^11^(Acm)]BM2(1–21)-Hmp-ADO_2_ (**9**), we were able to separate the two diastereomers which resulted from the racemic HMP building block used for the synthesis of **8–10**, allowing us to conclude that they are equally reactive. They also form similar and low amounts (∼4–12%, Table S4†) of hydrolyzed carboxyester. The reason for the lower hydrolysis rate observed in the ligation buffer C might be due to a lower water content in the buffer C in comparison to the buffer A, thus resulting in a faster reaction with the Cys-peptide.

### Desulfurization and Acm-group deprotection of BM2(1–51) (**12a**)

After the successful ligation of the BM2(1–51) proton channel through our HMP-strategy we focused on the back-transformation of the modifications, which we introduced with respect to our ligation strategy, namely cysteine at position 21 and an Acm protecting group at position 11. Hence, Cys21 was transformed into Ala through a desulfurization step, followed by cleavage of the Acm protecting group from Cys11.

Desulfurization of the Cys residues expands the number of possible ligation sites.^65^ This is especially of importance for peptides which have no naturally occurring or suitable Cys residues for the anticipated ligation strategy.

The desulfurization of Cys21 was achieved using the method introduced by Danishefsky *et al.*,^66^ which was optimized due to the insolubility of peptide **12a** in the desulfurization buffer.

Lastly, desulfurization was accomplished by the addition of the radical initiator VA-044 (2,2-azobis[2-(2-imidazolin-2-yl)propane]dihydrochloride) in the presence of reduced glutathione and an excess of tris(2-carboxyethyl)phosphine in a buffer/HFIP emulsion.

**Fig. 6:**
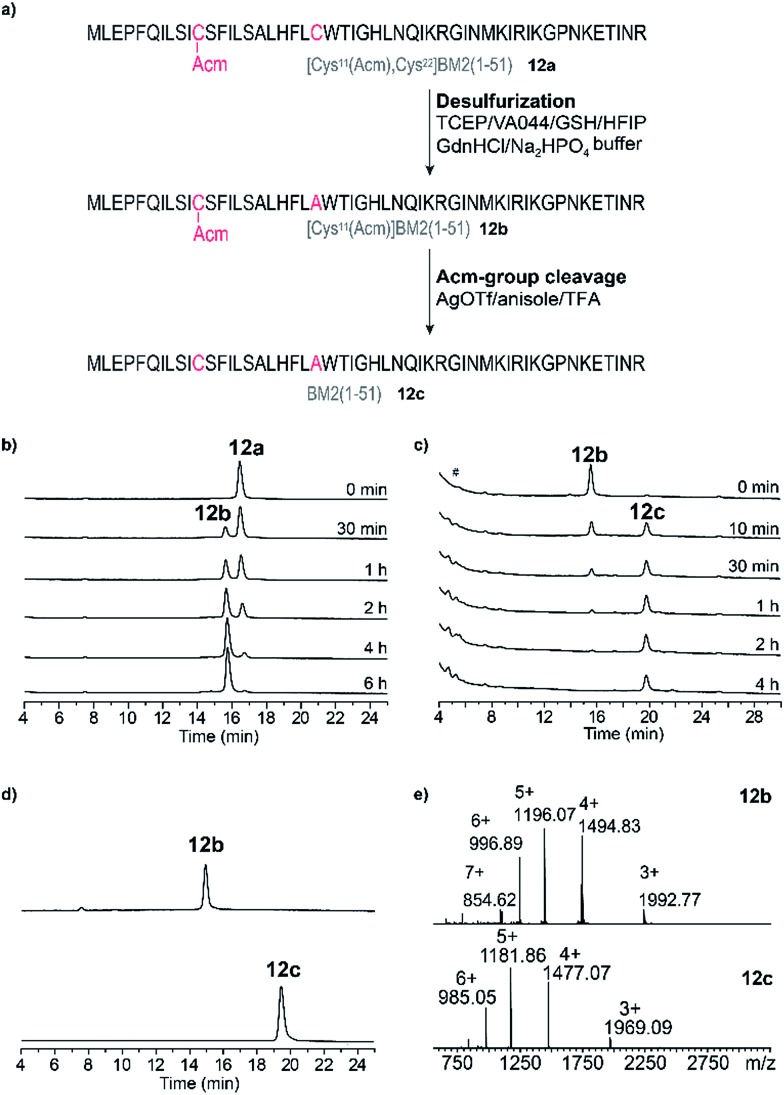
(a) Schematic representation of the post-NCL desulfurization (product **12b**) and cleavage of the Acm-group reactions leading to the final product BM2(1–51) (**12c**). Reaction conditions are highlighted for each step. (b) RP-HPLC elution profiles of the reaction mixture during desulfurization at different time points (directly after the reaction start (0 min)–6 h). (c) RP-HPLC elution profiles of the reaction mixture during cleavage of the Acm-group at different time points (directly after the reaction start (0 min)–4 h). # – injection peak. (d) RP-HPLC elution profiles of the purified products [(Cys^11^(Acm)]BM2(1–51)) (**12b**, upper trace) and the final product BM2(1–51) (**12c**, bottom trace). (e) ESI-MS spectra of [(Cys^11^(Acm)]BM2(1–51)) (**12b** upper spectrum) and BM2(1–51) (**12c**, bottom spectrum).

A nearly quantitative conversion of Cys to Ala was achieved for the peptide **12a** (within 6 h, Fig. 6b, Table S5†), yielding peptide **12b**. Interestingly, we found that by using this method, almost quantitative desulfurization can be accomplished for the soluble peptide **5** within 4 h (Fig. S7†). This result is similar to that described for small hydrophilic peptides in the literature, however the yield was reported to vary (80–99%) depending on the peptide sequence.^65^,^66^

Finally, for the cleavage of the Acm protection group from Cys11 to obtain **12c**, three different methods were tested using iodine oxidation,^67^,^68^ a silver salt,^69^–^74^ and DTNP.^75^–^78^ The DTNP approach, however, failed in our hands, probably because of the presence of Met and Trp in the primary structure of our peptide **12**.^79^ Using iodine oxidation we achieved only very poor yields (∼16%) of the final product **12c** (Fig. S8†). The Acm deprotection employing silver trifluoromethanesulfonate (AgOTf) resulted in a final yield of ∼60% of **12c** (Fig. 6c) after HPLC purification, which is comparable to the literature results for the hydrophilic peptide oxytocin.^70^ The peptide **12c** was finally purified (Fig. 6d and e, Table S5†) and subjected to CD analysis.

### Structural indications of the synthetic BM2(1–51)

**Fig. 7:**
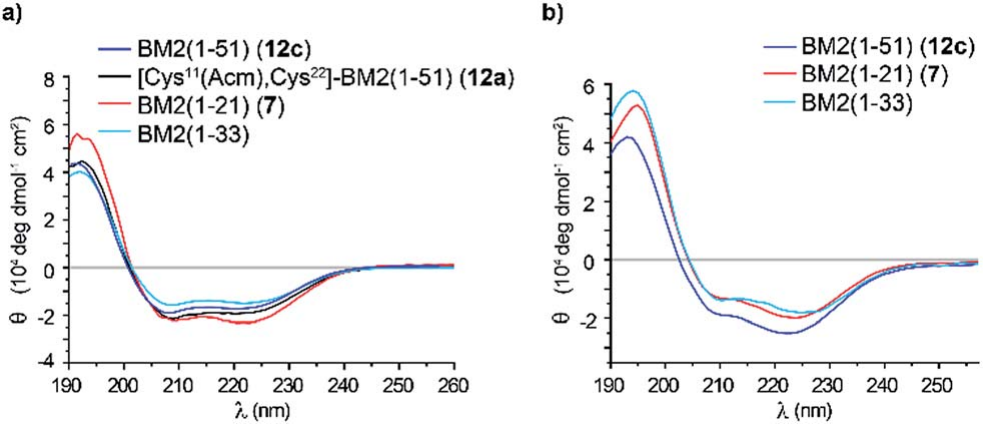
Structural indications for BM2(1–51) (**12c**). (a) The overlay of the CD spectra: BM2(1–51) (**12c**, blue), BM2(1–51) (**12a**, black), BM1(1–21) (**7**, red) and BM2(1–33) (cyan) in TFE. (b) CD spectra of BM2(1–51) (**12c**, blue), BM1(1–21) (**7**, red) and BM2(1–33) (cyan) in POPC lipid membrane.

In order to analyze whether the BM2(1–51) protein fragment (**12c**) would correctly fold, the final product **12c** was subjected to CD analysis. Additionally, the far UV CD spectra of BM2(1–21) (**7**) and BM2(1–51) (**12a**) at 20 °C were first measured in TFE (Fig. 7). All spectra showed the characteristic double minima at 209 nm and 223 nm, which are typical for an α-helical fold (Fig. 7). After deconvolution of the CD spectra, it was found that the peptides **12a** and **12c** and fragment **7** largely exist in an α-helical form (Table S7†), containing around 70% α-helix. As a reference, the BM2(1–33) fragment revealed around 60% α-helical content under these conditions. Moreover, deconvolution of the CD spectra of the BM2 fragments **7** and **12c** and BM2(1–33) in POPC (1-palmitoyl-2-oleoylphosphatidylcholine) revealed a similar α-helical content of approx. 60% for all three BM2 fragments.

These findings are also in line with the recent structural data from a solid-state NMR study of BM2(1–33), which indicated an almost fully α-helical structure.^54^ Consequently, BM2(1–51) (**12c**) appears to be structurally very similar compared to its shorter counterpart BM2(1–33), and this might indicate some ordered helical structure for the region between residues 34 and 44, which was predicted to be structurally disordered from a solution NMR study of the full length BM2.^54^

## Conclusion

In conclusion, the use of a removable solubilizing tag combined with an oxo-ester functional unit is an effective method for the synthesis and purification of highly hydrophobic peptide fragments. The oxo-ester peptide can be effectively used for the native chemical ligation with cysteine-containing peptide fragments, as demonstrated by the synthesis of the influenza virus B proton channel fragments BM2(17–35) and BM2(1–51). Moreover, our strategy allows the incorporation of various solubilizing tags into an oxo-peptide sequence, which was shown by the example of ADO, ADO_2_ or ADO-Lys_5_ tags. For the native chemical ligation, the Ala22 residue was replaced with Cys22, and Cys11 was protected with an Acm-group. Both modifications were fully reversible through desulfurization (99% yield) and Acm-group cleavage (60% yield) after the careful optimization of existing literature protocols for our hydrophobic peptides. Finally, circular dichroism analysis of the BM2 fragments, BM2(1–21) and BM2(1–51), in TFE and reconstituted in POPC revealed that the synthetic peptide exists mainly as an α-helical structure, similar to the recently characterized BM2(1–33), indicating that the structurally disordered region between residues 34–44 most likely possesses an α-helix structure.

The current study will provide an important alternative method to prepare highly hydrophobic peptides and proteins, allowing their synthesis in a multi-milligram scale, *i.e.* for future structural and binding studies.

## Experimental section

### Synthesis of 3-chloro-2-hydroxypropanoic acid

A 3 M solution of 3-chloro-1,2-propanediol (0.09 mol) in 30 mL nitric acid (conc.) was slowly heated to 80 °C until a vigorous reaction started. The temperature was kept at 80 °C for 20 min and then warmed-up to 100 °C for 30 min. Subsequently, the reaction solution was cooled to room temperature and neutralized with sodium bicarbonate. The product was extracted with diethyl ether, and the organic layer was dried over anhydrous sodium sulfate before the solvent was removed under reduced pressure. The residual solvent was evaporated to leave a viscous liquid. The product was precipitated in cold chloroform and filtered to obtain 7.4 g of 3-chloro-2-hydroxypropanoic acid (yield: 66%). NMR (DMSO-d_6_): *δ* 3.77 (2H, CH^β^), 4.30 (1H, CH^α^) (Fig. S1†).

### Synthesis of 2-hydroxy-3-(triphenylmethyl)thio-propanoic acid (Hmp(Trt)-OH)

A 1 M solution of 3-chloro-2-hydroxypropanoic acid (0.047 mol) in dry 1,2-dimethoxyethane was cooled to 0 °C, and sodium hydride (60% m/m dispersion in mineral oil, 0.047 mol) was added slowly. A solution of triphenylmethanethiol (0.05 mol) and sodium hydride (60% dispersion in mineral oil, 0.047 mol) in dry 1,2-dimethoxyethane was added dropwise, and the resulting reaction mixture was stirred for 4 h. Afterwards, the solvent was removed under reduced pressure yielding a yellow, solid residue, which was dissolved in a diethyl ether/water mixture (1 : 1). The aqueous phase was separated, washed with diethyl ether and acidified with 1 M hydrochloric acid. The product was extracted with ethyl acetate. Finally, the organic layer was dried over anhydrous sodium sulfate and the solvent was removed under reduced pressure until a highly viscous liquid was left. The liquid was solidified overnight in the refrigerator and the rest of the solvent was removed under reduced pressure until a slightly yellow powder was obtained. Yield: 11.08 g (98%). ^1^H-NMR (CDCl_3_): *δ* 2.63 (2H, CHβ2), 3.81 (1H, CH^α^), 7.12–7.38 (15H, Trt). ^13^C-NMR (CDCl_3_): *δ* 36.10 (CHβ2), 67.05, 68.96 (Ph_3_, CH^α^), 126.92, 127.16, 128.06, 129.54, 144.32 (phenyl), 176.40 (COOH). MS: 275.2 [TrtS]^–^, 362.9 [M – H]^–^ (Fig. S2†).

### Peptide synthesis

The Cys-containing peptide fragments [Cys^22^]BM2(22–35) (**5**) and [Cys^22^]BM2(22–51) (**11**) were synthesized on an automated peptide synthesizer (Liberty, CEM) according to a standard Fmoc-protocol on AmphiSpheres RAM resin (0.37 mmol g^–1^ loading size). The coupling reactions (15 min, double coupling) were performed using Fmoc-amino acids (4 equiv.), activated with 1-[bis(dimethylamino)methylene]-1*H*-1,2,3-triazolo[4,5-*b*]pyridinium 3-oxide hexafluorophosphate (HATU, 3.9 equiv.) and *N*-ethyl-*N*-(propan-2-yl)propan-2-amine (DIEA, 8 equiv.) in *N*,*N*-dimethylformamide (DMF) under microwave irradiation (50 °C, 35 W). Fmoc-deprotection was achieved by treating the peptide-resin with 20% piperidine in DMF under microwave irradiation (50 °C, 35 W), twice. All the deprotection and coupling steps were followed by intensive washing using DMF and dichloromethane (DCM).

The Hmp-containing model peptides (**1–4**) were synthesized manually on the resin. For the extended Hmp-containing peptides (**8–10**): the solubilizing tag (Lys_5_, ADO_2_), spacer (ADO), Hmp unit and the first two amino acids of the respective peptide fragment were coupled manually to the resin. The following amino acids were coupled (30 min, double coupling) using an automated peptide synthesizer (Liberty, CEM) applying identical conditions as those for peptides **5** and **11**. The *S*-acetamidomethyl (Acm) group was used as a side chain protection group for Cys11. For the peptides **8–10**, Fmoc-groups were removed with 20% 2-methylpiperidine (v/v) in DMF.

The amino acid Leu21 (Ile21) was coupled to the preceding Hmp-unit as described by Liu *et al.*^51^ Accordingly, the resin was re-swollen in dry oxolane (THF) and treated twice (double coupling) with a 0.2 M mixture of the amino acid (4 equiv.), triphenylphosphine (3.9 equiv.) and diethyl azodicarboxylate (40 wt% in toluene, 3.9 equiv.) for 2 h. Afterwards, the resin was washed intensively with THF.

Peptide cleavage and deprotection was accomplished in a mixture of 90% TFA, 5% water, 2.5% TIPS and 2.5% anisole for 3 h at room temperature. The crude peptides were precipitated in cold diethyl ether, centrifuged, and washed with diethyl ether.

### Peptide purification

The peptides **1–11** were purified by preparative RP-HPLC using a C18 column (MultoKrom 100-5, 250 × 20 mm, 100 Å pore diameter, and 5.0 μm particle size). The peptides **7–11**, **12a**, **12b** and **12c** were purified using a C4 column (MultoHigh Bio 300-5, 250 × 20 mm, 300 Å pore diameter, and 5.0 μm particle size). Water (containing 0.1% TFA, eluent A) and acetonitrile (containing 0.1% TFA, eluent B) were used as the HPLC eluent system. The RP-HPLC, MALDI-TOF MS and ESI-MS data of peptides **1–11**, **12a**, **12b** and **12c** are summarized in Table S2.†

### Native chemical ligation

The ligation experiments were performed in the ligation buffers (Buffer A, B and C, see Table S6†) at a 1 : 1.5 (n/n) ratio of the thioester-forming peptide (**1–4**, **8–10**, 1 mM) and Cys-peptide (5, 11, 1.5 mM). The ligation reaction was initiated by adjusting the pH of the ligation buffer to pH 7.0 or pH 7.5 with NaOH (1 M) depending on the used buffer (ESI Table S6†). All ligation experiments were performed under an N_2_ atmosphere.

The reaction progress was monitored by analytical RP-HPLC employing a C18 (150 × 4 mm, 100 Å pore diameter, and 3.0 μm particle size) or C4 column (100 × 4 mm, 120 Å pore diameter, and 2.1 μm particle size) with linear gradients of 15–45% eluent B over 30 min (for the ligation mixtures **1** + **5**, **2** + **5**, and **3** + **5**), 20–40% eluent B over 30 min (for the ligation mixture **4** + **5**) and 10–20% eluent B over 5 min followed by 20–70% eluent B over 20 min (for the ligation mixtures **8** + **11**, **9** + **11**, and **10** + **11**).

### Desulfurization of [Cys^11^(Acm), Cys^22^]BM2(1–51)

A 0.5 M tris(2-carboxyethyl)phosphine (TCEP) HCl solution in 6 M guanidinium hydrochloride (GdnHCl)/0.2 M disodium phosphate buffer was prepared. The pH of this solution was adjusted to 6.5 and reduced glutathione (GSH, 125 μL of a 160 mM solution in water) was added. The reaction was performed under a nitrogen atmosphere. 1.2 mg of the peptide [Cys^11^(Acm), Cys^22^]BM2(1–51) was dissolved in 2 mL HFIP and added to the reaction solution. Afterwards, 200 μL of a VA-044 solution (300 mM in water) was added and left to react for 6 h. The reaction progress was monitored by analytical RP-HPLC (C4 column 125 × 4 mm, 300 Å pore diameter, and 5.0 μm particle size) with a linear gradient of 45–70% eluent B over 30 min at a flow rate of 1 mL min^–1^. The reaction was stopped by transferring the lower layer into the 4-fold volume of 0.1% trifluoroacetic acid (TFA). The solution was freeze-dried and the peptide was isolated by preparative RP-HPLC using a C4 column (MultoHigh Bio 300-5, 250 × 20 mm, 300 Å pore diameter, and 5.0 μm particle size) with a linear gradient of 40–80% eluent B over 50 min at a flow rate of 10 mL min^–1^. Yield: 1.09 mg [Cys^11^(Acm)]BM2(1–51) (91%).

### Acm-group deprotection of [^11^Cys(Acm)]BM2(1–51) with silver trifluoromethanesulfonate

0.6 mg (1 equiv.) of [Cys^11^(Acm)]BM2(1–51) was dissolved in a solution of 7.7 mg silver trifluoromethanesulfonate (0.03 mM, 300 equiv.)/1.6 μL anisole (15 nM, 150 equiv.)/1 mL TFA, and left to react for 90 min at 4 °C under a nitrogen atmosphere. The reaction progress was monitored by analytical RP-HPLC employing a C4 column (125 × 4 mm, 300 Å pore diameter, and 5.0 μm particle size) with a linear gradient of 45–70% eluent B over 30 min at a flow rate of 1 mL min^–1^. Afterwards, the reaction was stopped by adding a two-fold excess of a DTT solution (6 M) in acetic acid/water (1 : 1). The solution was agitated for 2 h at 20 °C. Finally, the Ag-DTT supernatant was centrifuged, and the peptide was purified by preparative RP-HPLC using a C4 column (MultoHigh Bio 300-5, 250 × 20 mm, 300 Å pore diameter, and 5.0 μm particle size) with a linear gradient of 40–99% eluent B over 80 min at a flow rate of 10 mL min^–1^. Yield: 65% *via* HPLC.

### Circular dichroism (CD) spectroscopy

CD spectra were recorded at 190–260 nm (0.1 cm path-length cuvette, 20 °C) 30 min after the peptide (0.1 mg mL^–1^) was dissolved in TFE, buffer (0.2 M, Na_2_HPO_4_, pH 7.0), buffer/2,2,2-trifluorethanol (TFE, 2 : 1) and buffer/1,1,1,3,3,3-hexafluor-2-propanol (HFIP, 2 : 1). Lipid membrane samples were prepared in a degassed 10 mM Na_2_HPO_4_ buffer (pH 7.5) containing 0.5 mg mL^–1^ 1-Palmitoyl-2-oleoylphosphatidylcholine (POPC). Therefore, 15 eq. of POPC and 1 eq. of peptide were dissolved together in a small amount of TFE. The solvent was carefully removed using nitrogen gas, and the lipid/peptide mixture was dried under vacuum overnight. The dried residue was suspended in a degassed Na_2_HPO_4_ buffer (10 mM) and was shaken for 2 h at room temperature under an argon atmosphere. The sample was subjected to three freeze/thaw cycles and was finally extruded using a 1 μm polycarbonate membrane. Spectral deconvolution was performed using CDNN (Circular Dichroism analysis using Neural Networks) software.

## Conflicts of interest

There are no conflicts to declare.

## Supplementary Material

Supplementary information
