# Supplementary material for: Chemical synthesis of membrane proteins: a model study on the influenza virus B proton channel
Source: Chem Sci. 2018 Jan 22;9(8):2365–75. doi: 10.1039/c8sc00004b (PMC5897842; doi:10.1039/c8sc00004b)
Supplement: Supplementary file 1 [file SC-009-C8SC00004B-s001.pdf]

Electronic Supplementary Material (ESI) for Chemical Science.
This journal is © The Royal Society of Chemistry 2018

Electronic Supplementary Information for

Chemical synthesis of membrane proteins: a model study on the influenza virus B proton channel

Andreas C. Baumruck, Daniel Tietze, Lena Steinacker and Alesia A. Tietze*

*To whom correspondence should be addressed,
Email: a.tietze@tietze-lab.de

Table of Contents

Tables……………………………………………………………………………….3-6

Figures……………………………………………………………………………...7-18

Table S1. Cleavable solubility tags used to increase the solubility and NCL described in the literature

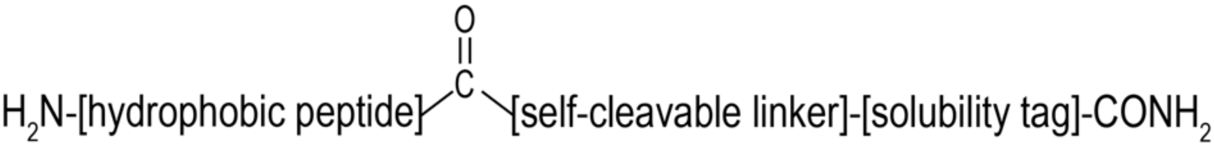

Peptide length	Peptide name	For purification	For ligation	Strategy	Linker	Solubility tag	Ref.
**12, 14**	dodecaalanine (Ala12) and chemotactic protein 10	x		Boc-SPPS	glycoamid ester[a]	$(GlyArg_4)_4$	[1]
**12-48**	model peptides	x		Fmoc-SPPS	4-Hmb[a]	$Gly(ArgGlyGly)_3$ $Gly(LysGly)_6$	[2]
**99**	HIV-1 protease		x	Boc-SPPS	3-mercaptopropionic acid[b]	$(Arg)_6$	[3]
**124**	ribonuclease A		x	Boc-SPPS	3-mercptopropionic acid[b]	$(Arg)_6$	[4]
**53**	insulin glargine	x		Fmoc-SPPS	HMBA[a]	$(Lys)_5$	[5]
**40**	cancer protein NY-ESO-1		x	Fmoc-SPPS, Boc-SPPS	HMBA[a]	$(Arg)_6$	[6]
**121**	diacylglycerol kinase (DAGK1-121)		x	Boc-SPPS	thioglycolic acid[b]	PPO/$(Arg)_6$	[7]
**79**	dengue 2 capsid protein C (DEN2C)		x	Boc-SPPS	3-mercptopropionic acid[b]	$(Arg)_6$	[8]
**32**	conotoxins	x		Fmoc-SPPS	PAM[c]	$(Lys)_4$	[9]
**42, 46**	Aß42 and Aß46	x		Fmoc-SPPS	no linker	$(Lys)_2$, $(Lys)_3$,$(Lys)_6$	[10]
**11**	Q11		x[e]	Fmoc-SPPS	Mmsb-OH[d]	$(Lys)_6$	[11]

HMBA – 4-hydroxymethylbenzoic acid
PPO – poly(ethyleneglycol)–polyamide
PAM – phenylacetamido
a – pH-sensitive
b – self-cleavable during NCL
c – cleavable by HF
d – reductive acidolysis using NH_4I
e – fragment condensation of two fragments

Table S2. Analytical data for peptides used in this study

	Peptide	Calculated M_w [g/mol]	Experimental M_w[b] [g/mol]	t_R[c] [min]
**1**	ALHFL-Hmp[a]	703.36	703.40	16.97/17.66[d]
**2**	ALHFL-Hmp-ADO[a]	848.43	848.51	17.15/17.65[d]
**3**	ALHFL-Hmp-ADO$_2$[a]	993.50	993.61	17.55/17.90[d]
**4**	ALHFL-Hmp-ADO-Lys$_5$[a]	1489.91	1489.02	6.62/6.97[d]
**5**	[Cys^{22}]BM2(22-35)	1637.90	1637.91	7.98[e]
**7**	BM2(1-21)-NH$_2$	2421.30	2422.35	12.55[f]
**8**	[Cys^{11}(Acm)]BM2(1-21)-Hmp	2596.33	2597.32/2597.38	12.34/12.46[g]
**9**	[Cys^{11}(Acm)]BM2(1-21)-Hmp-ADO$_2$	2886.48	2887.56/2887.56	9.95/10.36[h]
**10**	[Cys^{11}(Acm)]BM2(1-21)-Hmp-ADO-Lys$_5$	3382.89	3384.27	10.36[g]
**11**	[Cys^{22}]BM2(22-51)	3530.97	3531.34	8.19[i]

[a] Corresponds to abbreviation BM2(17-21);

[b] mass peaks detected as $[M+H]^+$;

[c] retention times of peptides **1-5** and **8**, **9** are given for both diastereomers, if applicable;

[d] HPLC conditions: 15–45 % eluent B in 30 min;

[e] HPLC conditions: 20–80 % eluent B in 20 min;

[f] HPLC conditions: 50 – 99 % eluent B in 15 min, detection was at 220 nm at a flow rate of 1 mL/min, C18 column;

[g] 10 – 20% eluent B in 5 min followed by 20 – 70% eluent B in 20 min, at a flow rate of 2 mL/min, C4 column;

[h] 40 – 80 % eluent B in 20 min, at a flow rate of 1 mL/min, C18 column;

[i] 20-40 % eluent B in 30 min at a flow of 1 mL/min, C18 column.

Table S3. Ligation results for model peptides

	Ligated fragments	[^{22}Cys(Acm)]BM2(17-35) (**6**), [%][a]	Hydrolyzed ester (ALHFL-C$^{\alpha}$OOH) [%][a]
**1+5**	ALHFL-Hmp + [Cys^{22}]BM2(22-35)	87	13
**2+5**	ALHFL-Hmp-ADO + [Cys^{22}]BM2(22-35)	86	14
**3+5**	ALHFL-Hmp-ADO$_2$ + [Cys^{22}]BM2(22-35)	85.4	14.6
**4+5**	ALHFL-Hmp-ADO-Lys$_5$ + [Cys^{22}]BM2(22-35)	84	16

[a] determined through integration of RP-HPLC chromatogram

Table S4. Ligation results for BM2 peptides in ligation buffers A, B and C

	Ligated fragments	[Cys^{11} (Acm)]BM2(1-51))(**12a**)/ hydrolyzed ester<br><br>([Cys^{11}(Acm)]BM2(1-21)-C$^{\alpha}$OOH)<br><br>in **ligation buffer A**[a]	[Cys^{11}(Acm)]BM2(1-51) (**12a**)/ hydrolyzed ester<br><br>([Cys^{11}(Acm)]BM2(1-21)-C$^{\alpha}$OOH)<br><br>in **ligation buffer B**[a]	[Cys^{11}(Acm)]BM2(1-51) **12a**)/ hydrolyzed ester<br><br>([Cys^{11}(Acm)]BM2(1-21)-C$^{\alpha}$OOH)<br><br>in **ligation buffer C** [%][a]
**8a+11**	[Cys^{11}(Acm)]BM2(1-21)-Hmp diastereomer A+ [Cys^{22}]BM2(22-51)	n.d.	n.d.	96/4
**8b+11**	[Cys^{11}(Acm)]BM2(1-21)-Hmp diastereomer B+ [Cys^{22}]BM2(22-51)	n.d.	n.d.	97/3
**9a+11**	[Cys^{11}(Acm)]BM2(1-21)-Hmp-ADO_2 diastereomer A + [Cys^{22}]BM2(22-51)	n.d.	65/45	96/4
**9b+11**	[Cys^{11}(Acm)]BM2(1-21)-Hmp-ADO_2 diastereomer B + [Cys^{22}]BM2(22-51)	n.d.	n.d.	96/4
**10+11**	[Cys^{11}(Acm)]BM2(1-21)-Hmp-ADO-Lys_5 + [Cys^{22}]BM2(22-51)	80/20	n.d.	88/12

[a] determined through integration of RP HPLC chromatogram

Table S5. Characterization of BM2(1-51) products 12a, 12b and 12c

	Peptide	Molecular formula	Calculated M_w [g/mol]	Experimental M_w[a] [g/mol]	t_R[b] [min]
**12a**	[Cys^{11}(Acm), Cys^{22}]BM2(1-51)	$C_{273}H_{448}N_{77}O_{67}S_4$	1202,85	1202,45	16.33
**12b**	[Cys^{11}(Acm)]BM2(1-51)	$C_{273}H_{447}N_{77}O_{67}S_3$	1196,24	1196,08	14.96
**12c**	BM2(1-51)	$C_{270}H_{442}N_{76}O_{66}S_3$	1182,02	1181,86	19.47

[a] mass peaks detected as $[M+5H]^{5+}$;

[b] HPLC conditions:45 – 70 % eluent B in 30 min, detection was at 220 nm at a flow rate of 1 mL/min, C4 column

Table S6. Ligation buffers used for study

Ligation buffer A	8 M Urea, 0.2 M disodium phosphate, 150 mM MPAA, 100 mM TCEP, pH 7.0
Ligation buffer B	Ligation buffer A : TFE (2:1), pH 7.5
Ligation Buffer C	3 M Urea, 75 mM disodium phosphate , 120 mM MPAA, 75 mM TCEP : HFIP (2:1), pH 7.5

MPAA = 4-mercaptophenylacetic acid, TCEP = tris(2-carboxyethyl)phosphine, TFE = 2,2,2-trifluoroethanol, HFIP = hexafluoro-2-propanol

Table S7. Deconvolution of CD spectra: (a) in TFE at wavelength 190-260 nm and **(b)** in liposome membrane POPC at wavelength 190-260 nm. Spectra deconvolution was performed using CDNN (Circular Dichroism analysis using Neural Networks) software.

a)

	**TFE**				
Sec. element	BM2(1-21) (7), %	BM2(1-51) (12a), %	BM2(1-51) (12c), %	AM2(22-46), %	BM2(1-33), %
**Helix**	**76.6**	**66.3**	**63.30**	**92.3**	**55**
Antiparallel	0.2	0.6	0.50	0	1.4
Parallel	2.7	3.5	4.80	0.9	5.1
Beta-Turn	10.2	12.0	11.60	7.7	13.4
Rndm. Coil	10.1	13.0	21.10	2.0	20
Total Sum	99.8	95.5	101.30	102.9	94.9

b)

	**Liposome membrane (POPC)**		
Sec. element	BM2(1-21) (7), %	BM2(1-51) (12c), %	BM2(1-33), %
**Helix**	**53.20**	**59.20**	**61.20**
Antiparallel	0.30	0.30	0.40
Parallel	9.40	6.80	5.70
Beta-Turn	11.10	11.00	11.40
Rndm. Coil	50.10	35.40	27.80
Total Sum	124.20	112.70	106.50

a)

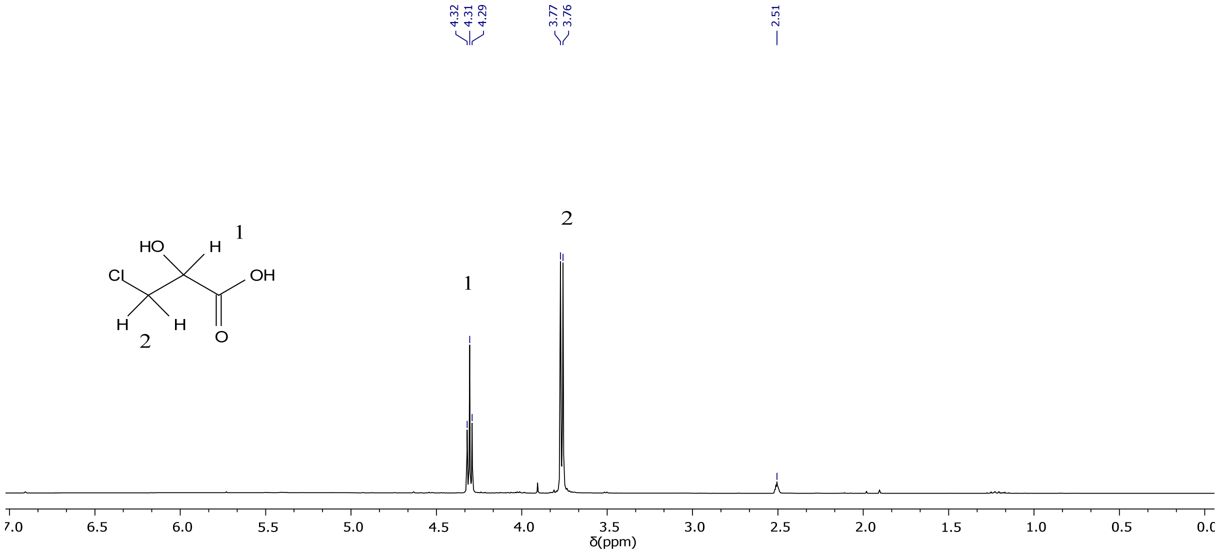

^{1}H NMR (300 MHz, DMSO-D6): δ = 4.32 – 4.29 (t, 2H), 3.77-3.76 (d, 1H), 2.51 (CDCl3).

b)

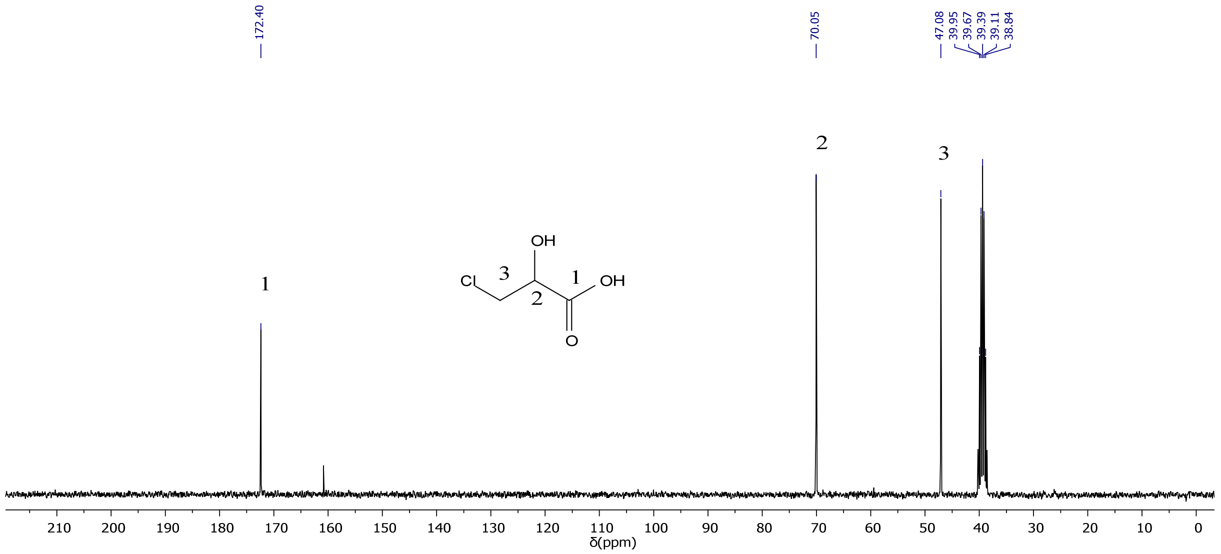

^{13}C NMR (75 MHz, DMSO-D6): δ = 172.40, 70.05, 47.08, 39.95-38.84 (DMSO).

Figure S1. Characterization of 3-chloro-2-hydroxypropanoic acid (Hmp). (**a**) ^{1}H-NMR spectrum of Hmp in DMSO-d6, (**b**) ^{13}C-NMR spectrum of Hmp in DMSO-d6

a)

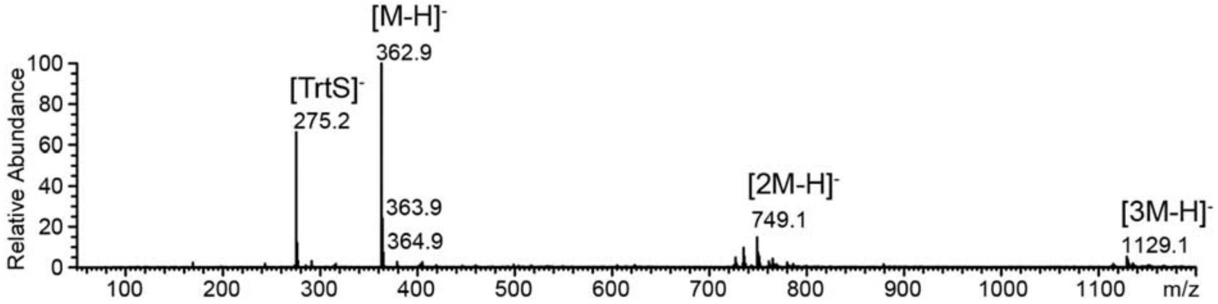

b)

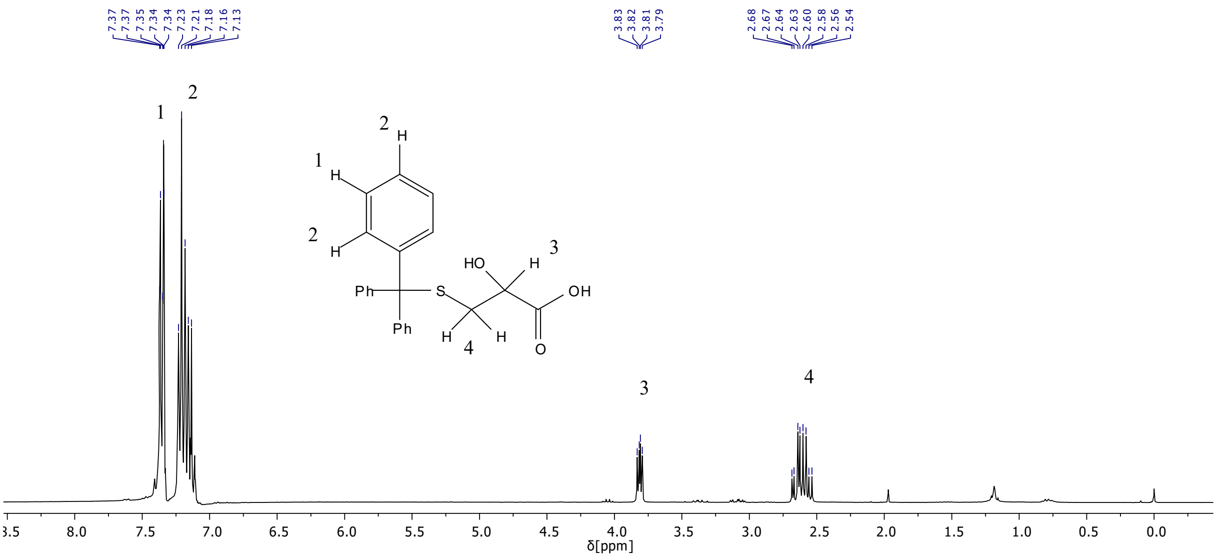

^{1}H NMR (300 MHz, $CDCl_3$): δ = 7.37 – 7.34 (m, 2H, H_{Ar}), 7.23 – 7.13 (m, 4H, H_{Ar}), 3.83 – 3.79 (m, 1H), 2.68 – 2.54 (m, 2H).

c)

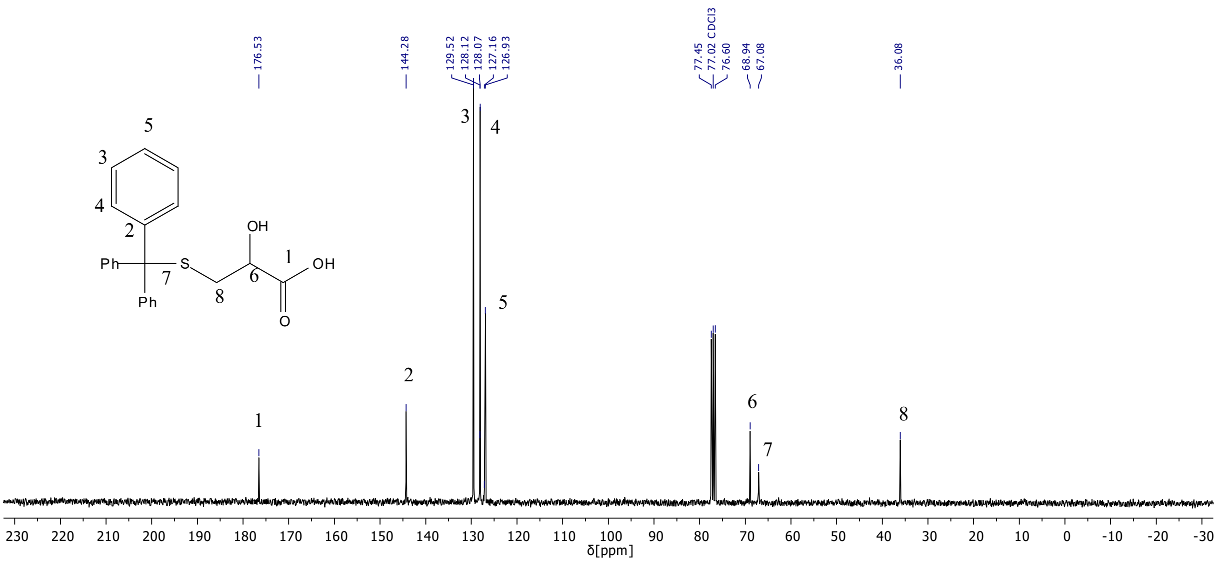

^{13}C NMR (75 MHz, $CDCl_3$): δ = 176.53, 144.28, 129.52, 128.12, 127.16, 68.94, 67.08, 36.08, 77.45-76.60 ($CDCl_3$).

Figure S2. Characterization of 2-hydroxy-3-(triphenylmethyl)thio-propanoic acid (Hmp(Trt)-OH). (**a**) ESI-MS spectrum in negative mode, (**b**) 1H-NMR spectrum of Hmp(Trt)-OH in $CDCl_3$, (**c**) 13C-NMR spectrum of Hmp(Trt)-OH in $CDCl_3$

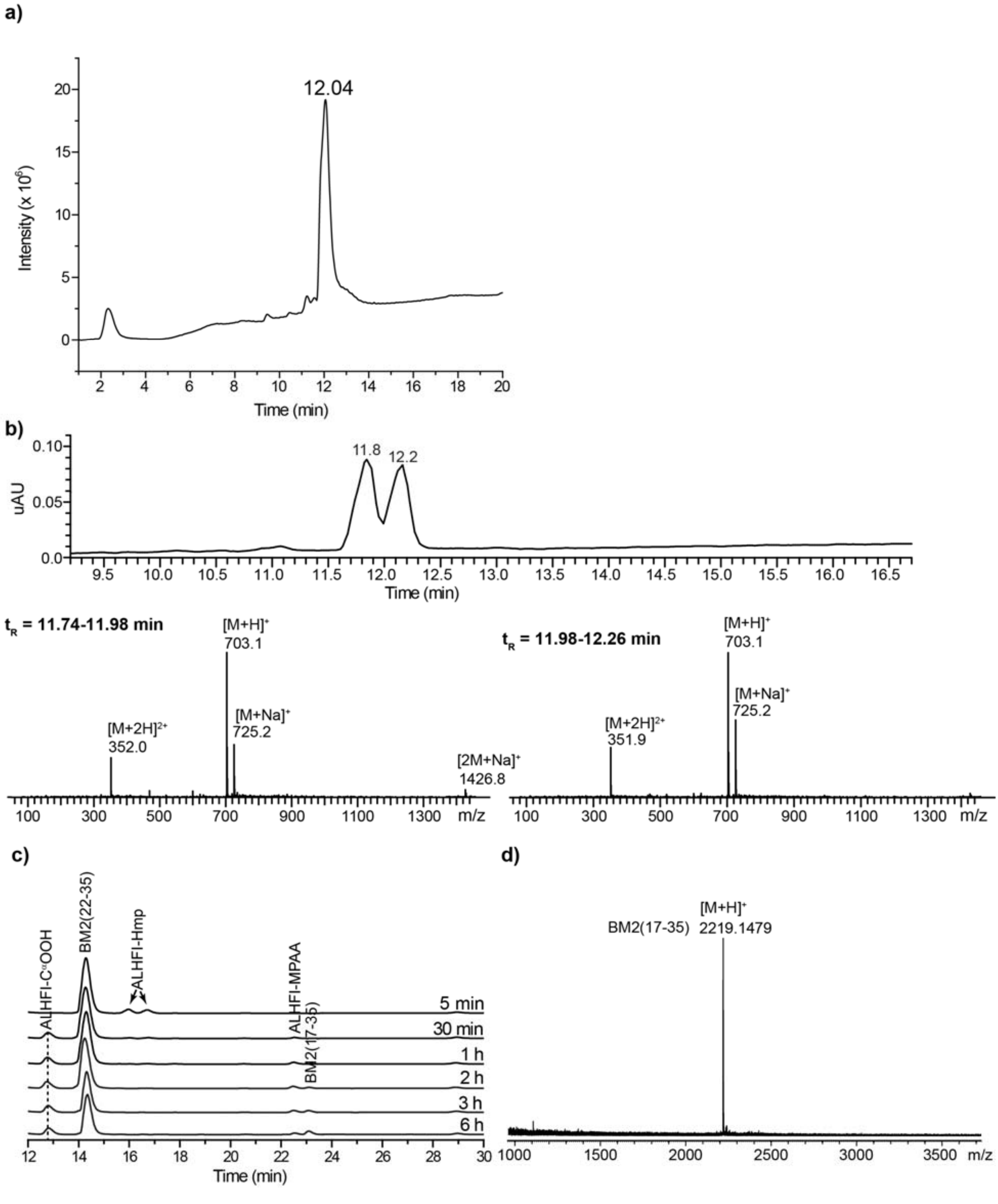

Figure S3. Characterization of ALHFI-Hmp peptide ligation behavior. (**a**) RP-HPLC elution profile of the crude product ALHFI-Hmp, Eluent A was 0.1% TFA in water, eluent B was 0.1 % TFA in acetonitrile and a gradient of 10–80 % eluent B in 20 min, detection was at 220 nm at a flow rate of 1 mL/min, C18 column (**b**) LC-MS of ALHFI-Hmp diastereomers A and B. , Eluent A was 0.1% TFA in water, eluent B was 0.1 % TFA in acetonitrile and a gradient of 20–80 % eluent B in 25 min using a C8 column and (**c**) RP-HPLC ligation monitoring of ALHFI-Hmp with BM2(22-35) at different time points and (**d**) MALDI-TOF MS of ligation product [Cys^{22}]BM2(17-35). HPLC conditions: 15–45 % eluent B in 30 min, detection was at 220 nm at a flow rate of 1 mL/min, C18 column

a)

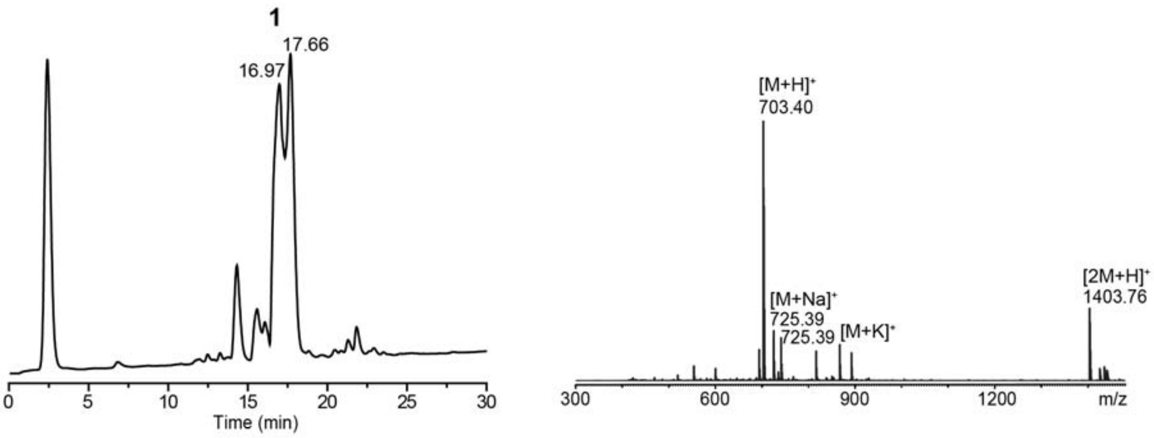

1
17.66
16.97
Time (min)
[M+H]+
703.40
[M+Na]+
725.39
725.39
[M+K]+
[2M+H]+
1403.76
m/z

b)

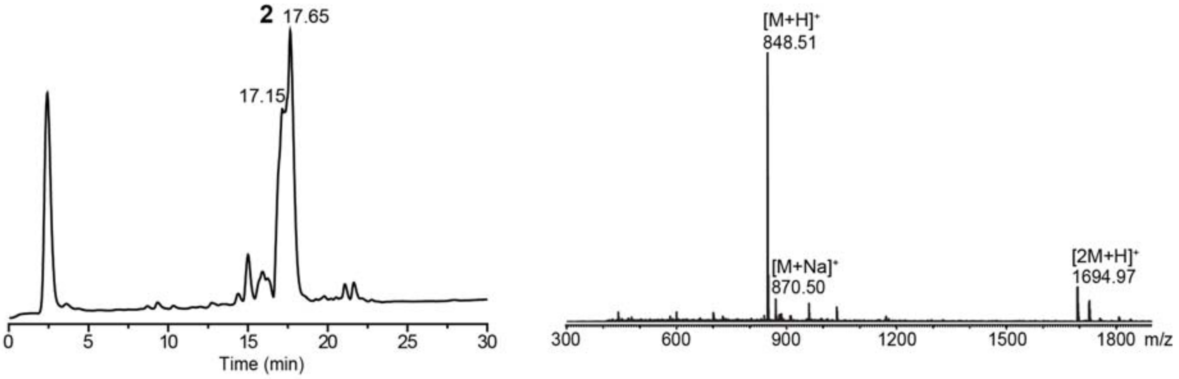

2
17.65
17.15
Time (min)
[M+H]+
848.51
[M+Na]+
870.50
[2M+H]+
1694.97
m/z

c)

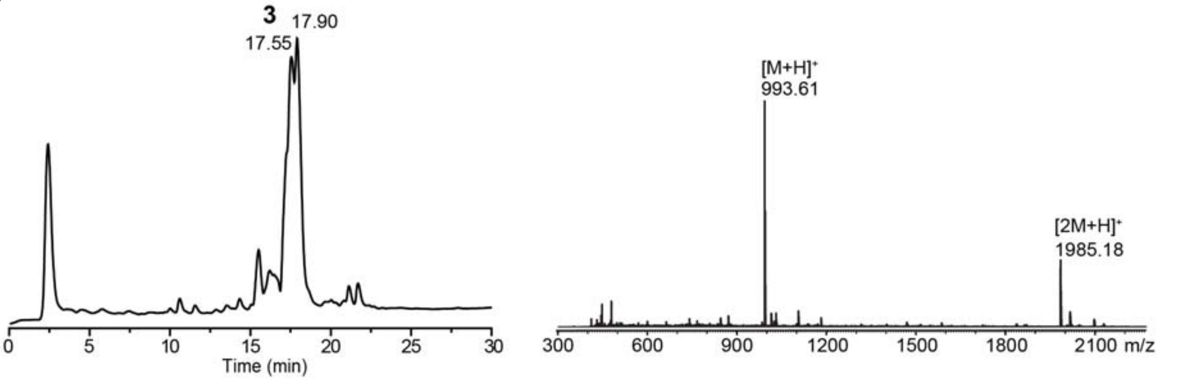

3
17.90
17.55
Time (min)
[M+H]+
993.61
[2M+H]+
1985.18
m/z

d)

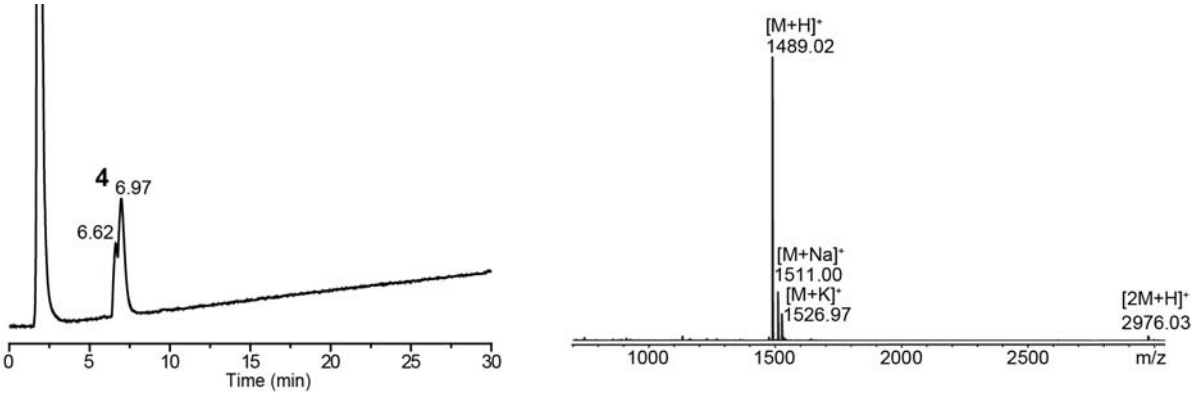

4
6.97
6.62
Time (min)
[M+H]+
1489.02
[M+Na]+
1511.00
[M+K]+
1526.97
[2M+H]+
2976.03
m/z

e)

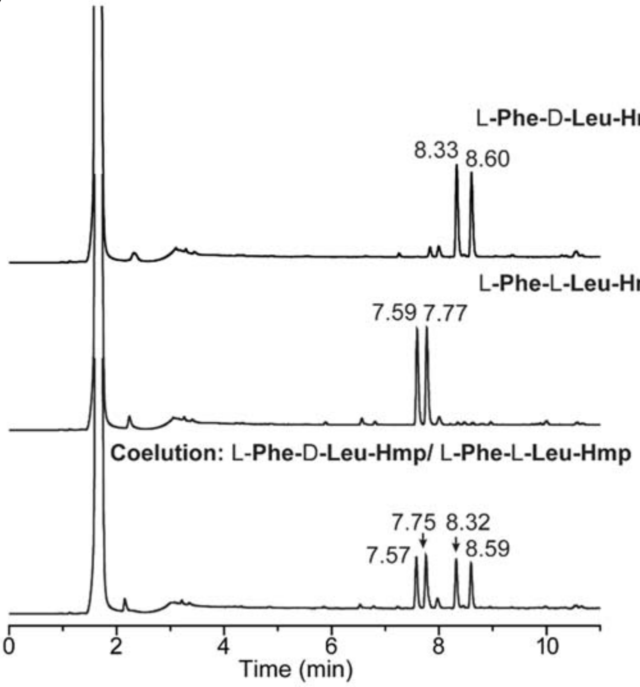

f)

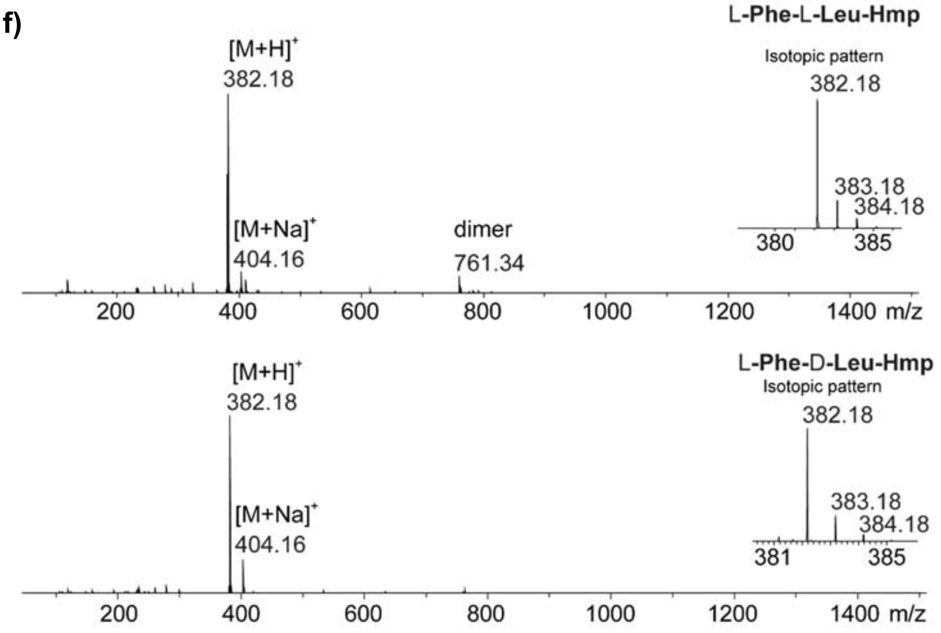

Figure S4. RP-HPLC and MALDI-TOF MS characterization of crude peptides and racemization behavior of Leu (directly attached to Hmp). (**a**) ALHFL-Hmp (**1**), (**b**) ALHFL-Hmp-ADO (**2**), (**c**) ALHFL-Hmp-ADO_2 (**3**) and purified (**d**) ALHFL-Hmp-ADO-Lys_5 (**4**). Eluent A was 0.1% TFA in water, eluent B was 0.1 % TFA in acetonitrile and a gradient for (**a-d**) 15–45 % eluent B in 30 min, detection was at 220 nm at a flow rate of 1 mL/min, C18 column; MALDI-TOF MS spectra for (**a-d**) were recorded at a reflector mode (500 shots/min) and as matrix α-cyano-4-hydroxycinnamic acid (HCCA) was used. (**e**) Stacked HPLC chromatograms of crude L-Phe-D-Leu-Hmp (upper chromatogram) and L-Phe-L-Leu-Hmp (middle) and and the coelution of two dipeptides. HPLC conditions: 0–80 % eluent B in 15 min, detection was at 220 nm at a flow rate of 1 mL/min, C18 column. (**f**) ESI MS spectra of L-Phe-L-Leu-Hmp and L-Phe-D-Leu-Hmp with isotopic patterns as insert.

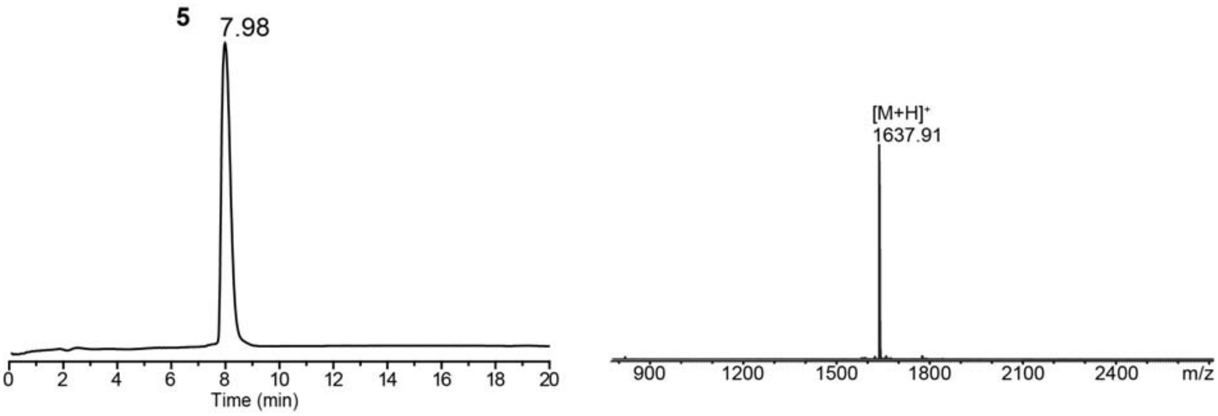

Figure S5. RP-HPLC and MSLDI-TOF MS characterization of purified [^{22}Cys]BM2(22-35) Cys-fragment (5). Eluent A was 0.1% TFA in water, eluent B was 0.1 % TFA in acetonitrile and a gradient: 20–80 % eluent B in 20 min, detection was at 220 nm at a flow rate of 1 mL/min, C18 column. MALDI-TOF MS spectra for (A-D) were recorded at a reflector mode (500 shots/min) and as matrix α-cyano-4-hydroxycinnamic acid (HCCA) was used.

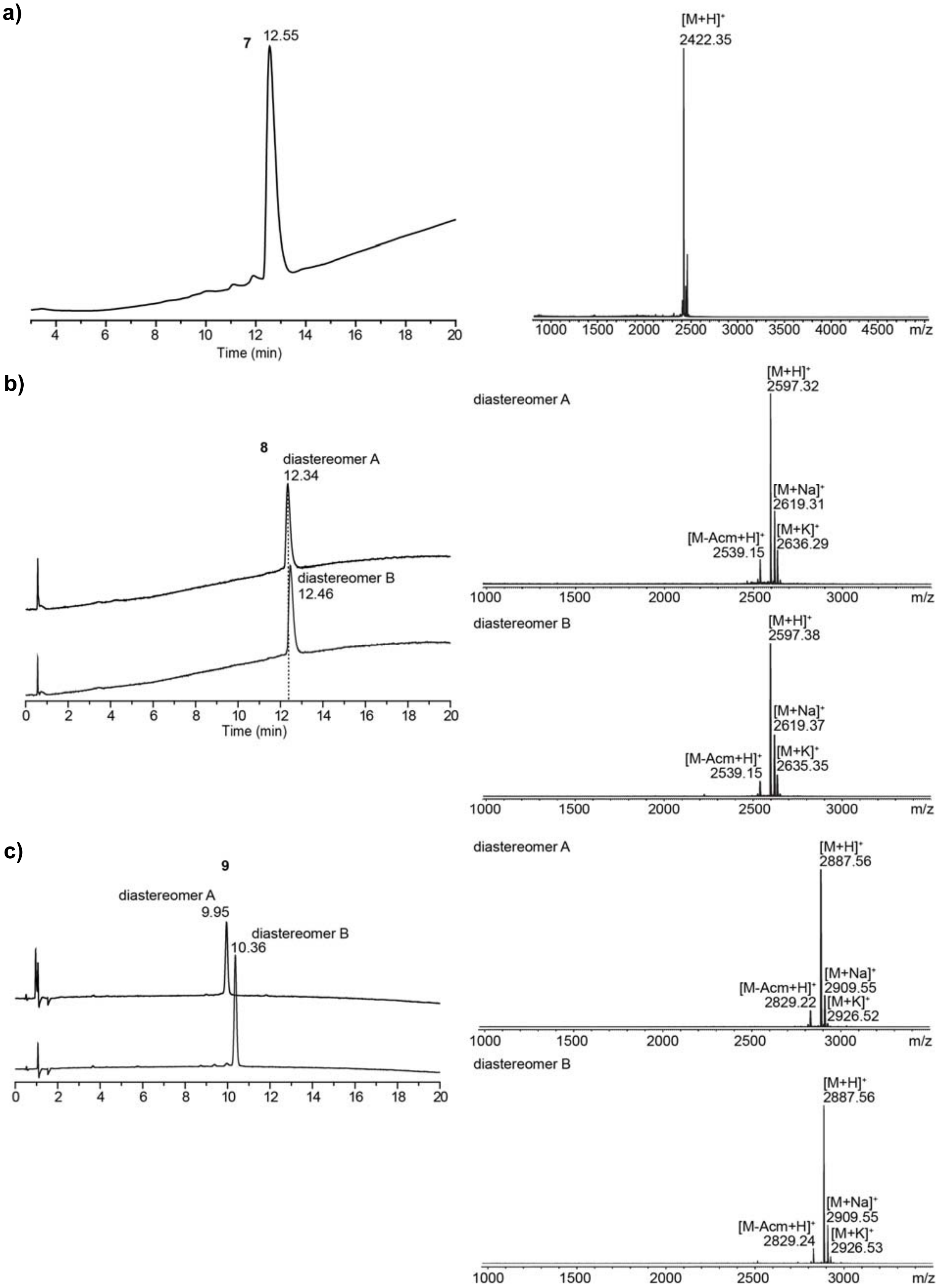

a)
7
12.55
Time (min)
[M+H]+
2422.35
m/z
b)
8
diastereomer A
12.34
diastereomer B
12.46
Time (min)
[M+H]+
2597.32
[M+Na]+
2619.31
[M+K]+
2636.29
[M-Acm+H]+
2539.15
[M+H]+
2597.38
[M+Na]+
2619.37
[M+K]+
2635.35
[M-Acm+H]+
2539.15
m/z
c)
9
diastereomer A
9.95
diastereomer B
10.36
[M+H]+
2887.56
[M+Na]+
2909.55
[M-Acm+H]+
2829.22
[M+K]+
2926.52
[M+H]+
2887.56
[M+Na]+
2909.55
[M-Acm+H]+
2829.24
[M+K]+
2926.53
m/z

d)

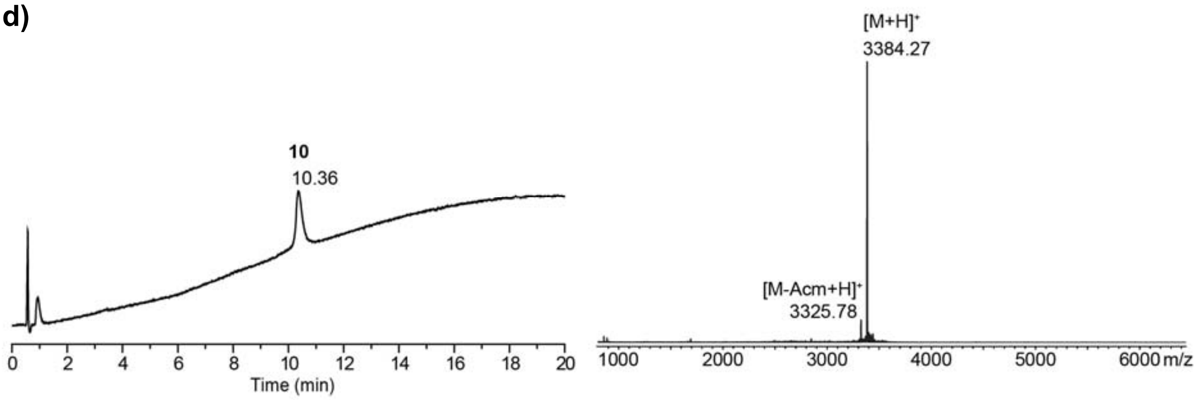

Figure S6. RP-HPLC and MALDI-TOF MS characterization of purified (**a**) [Cys[11](Acm)]BM2(1-21) (**7**), (**b**) [Cys[11](Acm)]BM2(1-21)-Hmp diastrereomer A/diastereomer B (**8a/8b**), (**c**) [Cys[11](Acm)]BM2(1-21)-Hmp-ADO_2 diastrereomer A/ diastereomer B (**9a/9b**), (**d**) [Cys[11](Acm)]BM2(1-21)-Hmp-ADO_2-Lys_5 (**10**). Eluent A was 0.1% TFA in water, eluent B was 0.1 % TFA in acetonitrile and a gradient: (**a**) 50 – 99 % eluent B in 15 min, detection was at 220 nm at a flow rate of 1 mL/min, C18 column; (**b**) 10 – 20% eluent B in 5 min followed by 20 – 70% eluent B in 20 min, at a flow rate of 2 mL/min, C4 column; (**c**) 40 – 80 % eluent B in 20 min, at a flow rate of 1 mL/min, C18 column; (**d**) 10 – 20 % eluent B in 5 min followed by 20 – 70% eluent B in 20 min, at a flow rate of 2 mL/min, C4 column. MALDI-TOF MS spectra for (**a-d**) were recorded at a reflector mode (500 shots/min) and as matrix α-cyano-4-hydroxycinnamic acid (HCCA) was used.

a)

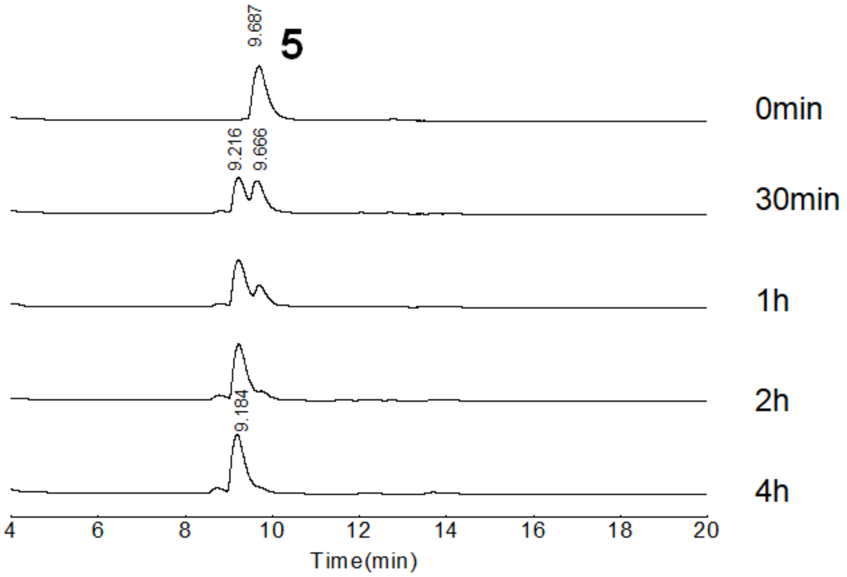

b)

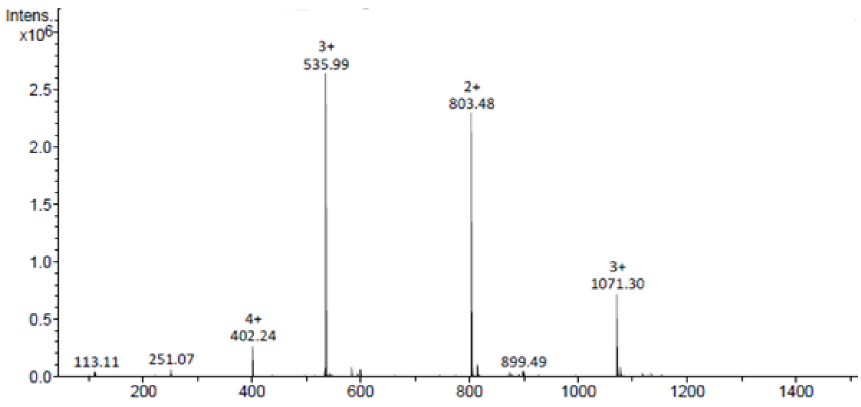

Figure S7. Desulfurization of peptide [Cys^{22}]BM2(22-35) (5). (**a**) RP-HPLC elution profile of the reaction mixture at different time points: 0 min (directly after reaction start) – 4 h. Initial peptide eluting at t_R = 9.68 min and reaction product [^{22}Ala]BM2(22-35) at t_R = 9.18 min. Eluent A was 0.1% TFA in water, eluent B was 0.1 % TFA in acetonitrile at gradient: 15 – 45 % eluent B in 30 min, detection was at 220 nm at a flow rate of 1 mL/min, C18 column. (**b**) ESI-MS of the reaction mixture after 4 h of reaction time. Following mass peaks belong to the product [Ala^{22}]BM2(22-35): 402.24 $[M+4H]^{4+}$, 535.99 $[M+3H]^{3+}$, 803.48 $[M+2H]^{2+}$. The mass peak at 1073.30 $[M+3H]^{3+}$belongs the **dimerized peptide 5** (BM2(22-35)[Cys^{22}]–[Cys^{22}]BM2(22-35)).

a)

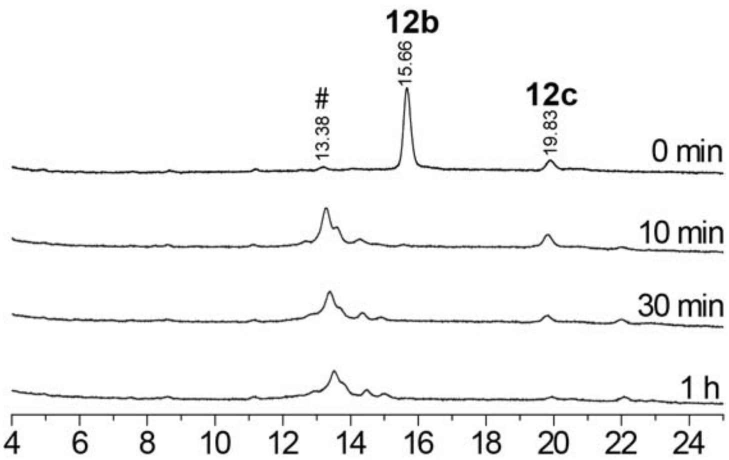
12b
15.66
#
13.38
12c
19.83
0 min
10 min
30 min
1 h
4 6 8 10 12 14 16 18 20 22 24

b)

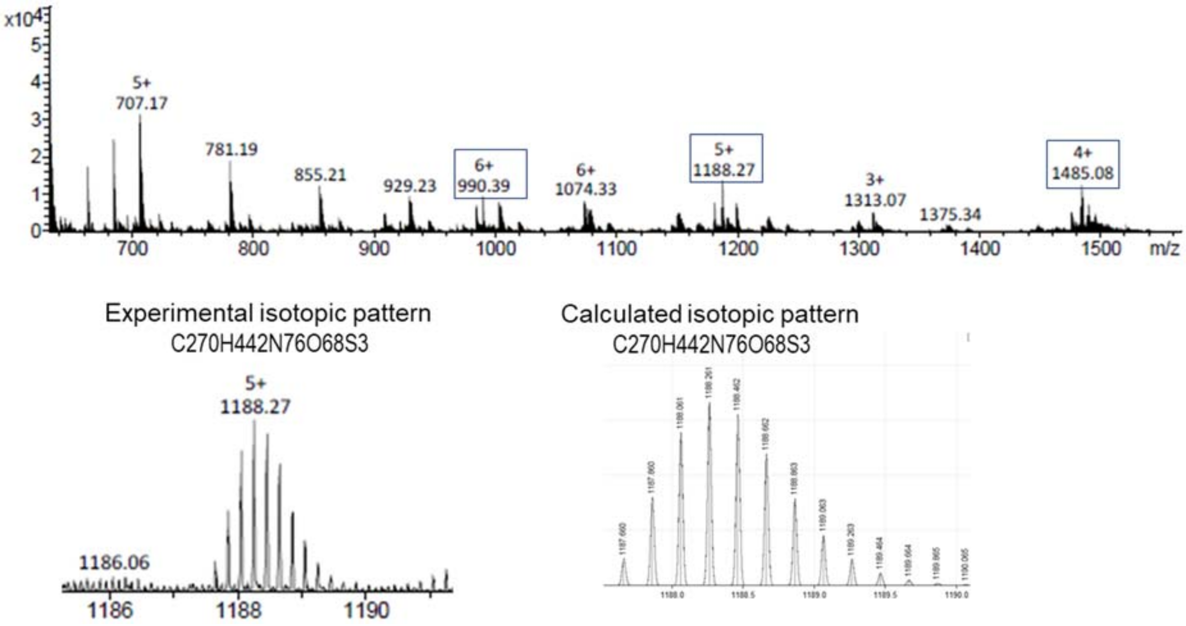
x10^4
5+
707.17
781.19
855.21
929.23
6+
990.39
6+
1074.33
5+
1188.27
3+
1313.07
1375.34
4+
1485.08
700 800 900 1000 1100 1200 1300 1400 1500 m/z
Experimental isotopic pattern
C270H442N76O68S3
5+
1188.27
1186.06
1186 1188 1190
Calculated isotopic pattern
C270H442N76O68S3

c)

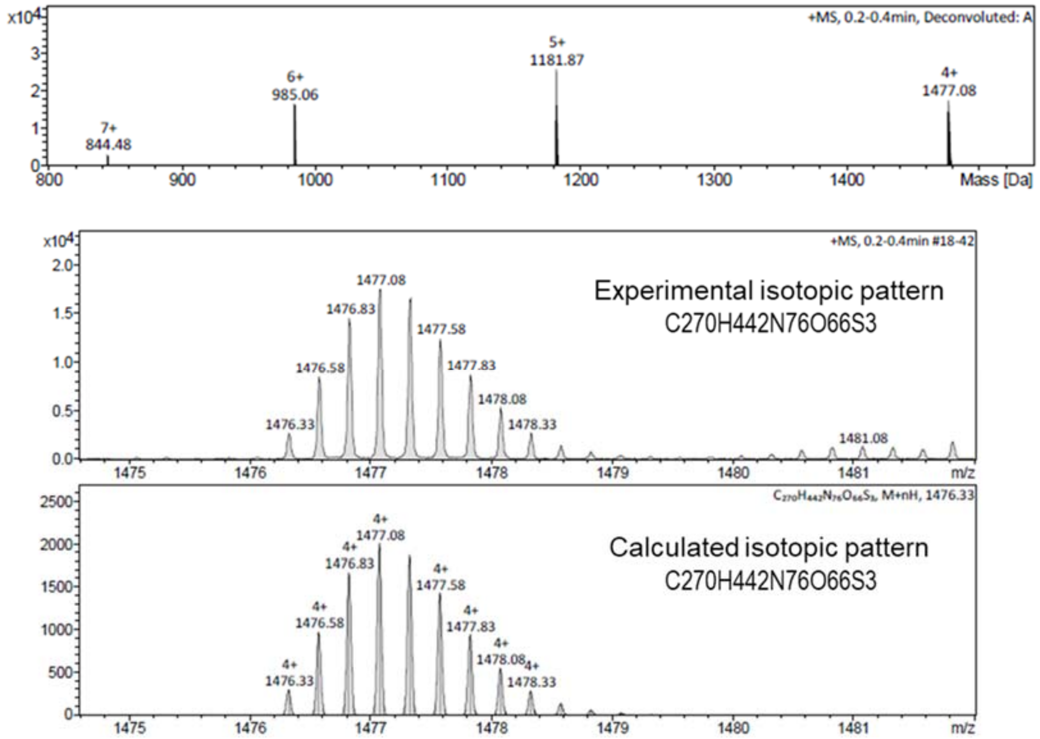

Figure S8. (a) RP-HPLC elution profiles of Acm-group cleavage from [Cys^{11}(Acm)]BM2(1-51) (**12b**) in iodine/acetic acid solution at different time points 0 min (directly after reaction start) – 1 h. Reaction products are highlighted as **12c** and #. According to peak integration the yield of product **12c** after 10 min is ~ 16 %. HPLC conditions: 45 – 70 % eluent B in 30 min, detection was at 220 nm at a flow rate of 1 mL/min, C4 column (**b**) ESI-MS of isolated reaction products t_R=13.38 min: # - BM2(1-51) where sulfur group of cysteine is present as sulfone and corresponding experimental and calculated isotopic patterns. (**c**) ESI-MS of deconvoluted product t_R=19.83 min: BM2(1-51) (**12c**) and corresponding experimental and calculated isotopic patterns.

References

1. D. R. Englebretsen and P. F. Alewood, *Tetrahedron Lett.*, 1996, **37**, 8431-8434.
2. C. T. Choma, G. T. Robillard and D. R. Englebretsen, *Tetrahedron Lett.*, 1998, **39**, 2417-2420.
3. E. C. Johnson, E. Malito, Y. Shen, D. Rich, W.-J. Tang and S. B. Kent, *J. Am. Chem. Soc.*, 2007, **129**, 11480-11490.
4. D. J. Boerema, V. A. Tereshko and S. B. Kent, *Pept. Sci.*, 2008, **90**, 278-286.
5. M. A. Hossain, A. Belgi, F. Lin, S. Zhang, F. Shabanpoor, L. Chan, C. Belyea, H.-T. Truong, A. R. Blair and S. Andrikopoulos, *Bioconjugate Chem*, 2009, **20**, 1390-1396.
6. P. W. Harris and M. A. Brimble, *Pept. Sci.*, 2010, **94**, 542-550.
7. S. Lahiri, M. Brehs, D. Olschewski and C. F. Becker, *Angew. Chem. Int. Ed.*, 2011, **50**, 3988-3992.
8. C. Zhan, L. Zhao, X. Chen, W.-Y. Lu and W. Lu, *Bioorg. Med. Chem.*, 2013, **21**, 3443-3449.
9. S. Peigneur, M. Paolini-Bertrand, H. Gaertner, D. Biass, A. Violette, R. Stocklin, P. Favreau, J. Tytgat and O. Hartley, *The Journal of biological chemistry*, 2014, **289**, 35341-35350.
10. S. Chemuru, R. Kodali and R. Wetzel, *Pept. Sci.*, 2014, **102**, 206-221.
11. M. Paradis-Bas, J. Tulla-Puche and F. Albericio, *Org. Lett.*, 2015, **17**, 294-297.
